# The Evolution of Musical Diversity: The Key Role of Vertical Transmission

**DOI:** 10.1371/journal.pone.0151570

**Published:** 2016-03-30

**Authors:** Sylvie Le Bomin, Guillaume Lecointre, Evelyne Heyer

**Affiliations:** 1 Eco-Anthropologie et Ethnobiologie, UMR 7206 CNRS, MNHN, Université Paris Diderot, Sorbonne Universités, Muséum national d’Histoire naturelle, Musée de l’Homme, Paris, France; 2 Department “Systématique et Evolution”, UMR 7205 CNRS-MNHN-UPMC-EPHE “Institut de Systématique, Evolution et Biodiversité”, Sorbonne Université, Muséum national d’Histoire naturelle, Paris, France; Max Planck Institute for Human Cognitive and Brain Sciences, GERMANY

## Abstract

Music, like languages, is one of the key components of our culture, yet musical evolution is still poorly known. Numerous studies using computational methods derived from evolutionary biology have been successfully applied to varied subset of linguistic data. One of the major drawback regarding musical studies is the lack of suitable coded musical data that can be analysed using such evolutionary tools. Here we present for the first time an original set of musical data coded in a way that enables construction of trees classically used in evolutionary approaches. Using phylogenetic methods, we test two competing theories on musical evolution: vertical versus horizontal transmission. We show that, contrary to what is currently believed, vertical transmission plays a key role in shaping musical diversity. The signal of vertical transmission is particularly strong for intrinsic musical characters such as metrics, rhythm, and melody. Our findings reveal some of the evolutionary mechanisms at play for explaining musical diversity and open a new field of investigation in musical evolution.

## Introduction

For more than a century, a central question has engaged anthropologists interested in the study of cultural evolution: are cultural traits transmitted primarily from ancestral to descendant populations (vertical transmission) or between neighboring populations (horizontal transmission), or do they emerge as independent evolution? [1] Although music is a key component of culture, the question of vertical versus horizontal transmission in music has never been tested. Recently two studies compared musical and genetic distances among populations. Brown et al, 2013 [2] show that music distances, based on songs, correlate with genetic distances among Taiwan populations. Pamjev et al, 2012 [3] also found such correlation as significant among Eurasian populations. But these correlations can occur because of either vertical or horizontal transmissions of both genes and music.

On the other hand, methods developed in evolutionary biology have been successfully applied to cultural data mainly in the field of linguistics [4, 5, 6, 7] but also marginally applied to some other material cultural data [8, 9, 10, 11, 12, 13, 14, 15] and also the evolution of two musical instruments [16].

Applying these methodologies to music, we test the existence of vertical against horizontal transmission in this key component of culture.

Cladograms represent “whom is sharing what with whom” in a hierarchical manner. They are used in evolutionary biology and systematics where hierarchy among shared traits is postulated to be consequence of common ancestry. However this postulate is not required for the use of cladograms. They can either be used to test for hierarchy (whatever the cause), or can just be used as tools to measure the ratio of horizontal exchange versus vertical inheritance. If the resulting parsimonious tree has a high consistency level [17] the most likely explanation is that the character states are transmitted vertically along the branches. We may otherwise have to consider that character states are shared through other processes, possibly through horizontal diffusion or convergent evolution.

For testing this vertical transmission, we build a musical dataset that consist of the coding of 58 musical patrimonies from Gabon. Patrimonies were analysed through 700 musical pieces collected in Gabon between 2000 and 2011. All musical traits were coded with exogenous and endogenous characters, including metrics, scales, polyphonic processes, instruments and repertoires. To our knowledge, this is the largest corpus of encoded music ever published from first-hand fieldwork data. It comprises in total 322 characters, each coded with 2 to 22 distinct character states.

## Material and Methods

### Sample

**Fig 1:**
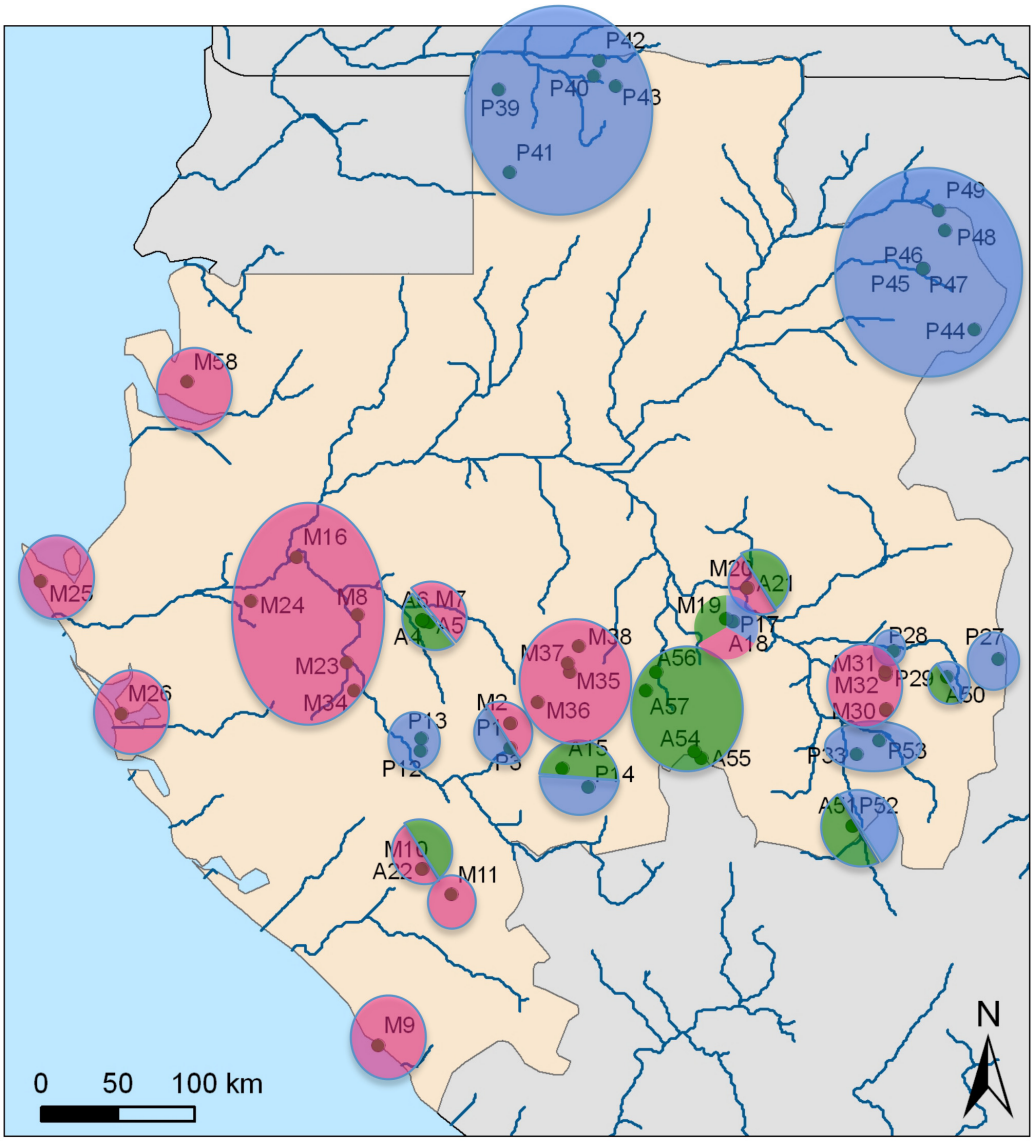
Geographical distribution of musical patrimonies with descent rule system identification of the ethnic group. Blue corresponding to patrilineal groups, pink to matrilineal groups and green to ambivalent groups). Codes (e.g. M9) refer to a patrimony described in Table 1. Map created by Sylvie Le Bomin from fieldwork data. (Source adapted from Institut national de cartographie du Gabon).

Data was collected in groups of farmer and hunter-gatherer Bantu (28) and Ubanguian (1) speakers from Gabon according to a convention and field work authorizations from the Omar Bongo University in Gabon. Our sampling is representative of rural areas in Gabon and does not include the capital area. We have no sample from the middle of the country where human settlements are scarce (Fig 1).

The data matrix (S1 Dataset) was built using 322 characters identified in previous studies on the characterization and categorization of musical practices in Central Africa [18, 19, 20, 21]. Each musical patrimony **(a set of musical features shared by a population)** consists on average of 6 repertoires, from which 2 to 3 pieces have been analysed. In total 700 pieces were analysed in the 58 patrimonies.

Values were determined using different methods according to the nature of the character: repertoire and musical instruments were collected by intensive and extensive questionnaire and direct observations; form, vocal techniques and polyphonic processes values were identified by listening pieces from each repertoire during different types of performances; metric, rhythmic and melodic values were deduced from analyses of musical transcriptions obtained with the help of the re-recording method. This is an analytical tool used in ethnomusicology: each instrument/voice is recorded independently. In case where several instruments/voices play the same melody, only one is recorded. Each musician listens to the musical part that he uses as a melodic or rhythmical reference at the same time as he plays [18]. A presence/absence data matrix was built for the 322 characters for 58 musical patrimonies collected across 75 localities and among 29 distinct ethnonymic entities (S1 Dataset). The data matrix was constructed following hypotheses of primary homologies [22]i.e. hypotheses of homology that have not yet been tested through a phylogenetic tree. Homologies confirmed by the tree are called secondary homologies.

Our cultural sample spans the diversity of rules of descent that can be found in Gabon: matrilineal (Ma), patrilineal (Pa), and ambivalent [23]. This is a social system of transmission of filiation; in our case it can be either patrilineal or matrilineal if the transmission comes from the father or from the mother respectively. Ambivalent is the case were both systems co-exist. Popular discourse admits two geographical areas: a patrilineal territory north of Ogooue River, and a southern matrilineal territory. The latter, however, is heterogeneous and integrates patrilineal ngom groups and ambivalent bongo groups for which the descent system is not yet clearly understood.

### Musical patrimonies as cultural taxa

The basic operational taxonomic units (OTU) considered here are musical patrimonies. Patrimonies were named after the endogenous ethnonym of the group and location where they were collected. Indeed, earlier studies have shown that there exists great regional variability among different groups scattered geographically [24, 25, 26]. Groups were identified according to their endogenous ethnonym and confirmed using a pluridisciplinary approach that combined literature review of anthropological studies and linguistic and genetic data. Such a pluridisciplinary approach is plainly justified as, for example, pygmy groups in Gabon share a genetic common ancestor [27] while there is no pygmy linguistic family [28, 29]. On the other hand, genetic studies are not able to distinguish bantu groups in Western Central Africa [27] while linguistics identify as many distinct groups as families [30, 31, 32].

### Musical parameters as characters

A patrimony is defined as a set of everything that is necessary for performing music in a given population. As music is performed in specific contexts, patrimonies also encompass contextual (e.g. circumstances, functions) and linguistic (e.g. names of repertoires and instruments) data.

We identified three categories of characters, distinguished according to previous systematic and categorization works [18, 19].

- repertoire: set of pieces including circumstance and social or symbolic implicit information;
- performativ: polyphonic process, form, instruments and vocal techniques;
- intrinsic: metrics, rhythm and melodic.

The repertoire is made up of sets of pieces (sang or not), most often named in the vernacular language. A repertoire is always performed for specific social circumstances and plays social and/or symbolic functions.

The polyphonic process was assessed in the vocal performance of each piece. It defines the superposition process of the different melodic parts. The form character defines how several singers exchange melodic-rhythmic material during a piece. The character was coded differently whether the alternative singers answer to the leading singer or repeat what he sings. Musical instruments, each of which is specifically named in the local vernacular language, were described according to organological classification [33].

The vocal technique is defined by which of the four phonatory systems is chosen to perform vocal music. These four phonatory systems refer to the different ways the two muscles in the larynx are activated [34].

For values of repertoire, musical instruments, vocal technique, polyphonic process and form presence was coded by 1, absence by 0. Missing data were coded with a question mark.

All sound parameters (metric, rhythmic, melodic) were considered as intrinsic characters. In musicology, the notion of time relies on two distinct principles, the metrics and rhythm, each of which is made up of constitutive parameters. The metrics gives the time standard [18]. It is made of beat, subdivision mode (binary or ternary), and period. The rhythmic pattern is composed of a succession of rhythmic cells, each one beat long and considered as a character.

Melodic character is represented by musical scale [18]. In oral tradition music, scales are determined through pieces transcriptions.

Almost all intrinsic characters are found in every population, but in each population they can be found in different repertoires. These intrinsic characters are distinctive traits which are used in ethnomusicological categorization [19]. We therefore used a specific repertoire coding system to mark the presence of the intrinsic character in each patrimony. The result is a multi-state coding [35] of these characters: each state is the repertoire in which the given intrinsic character is found. For example a triplet can be found in repertoire “mungala” and repertoire “lisimbu”. It will be coded {a,b}, then if in another patrimony it is found in the “lisimbu” repertoire and “nzobi” repertoire, it will be coded {b,c}.

Then during the parsimony reconstruction, for this character, there will be a similarity among these two patrimonies because they share {b} for this character. This gives a different weight to the character than with a binary coding where each character would be split in three “characters” and the codes would be (1,1,0) for the first patrimony and (0,1,1) for the second patrimony. This is in accordance with our current knowledge on music systematics, where having a shared state for a given character within a repertoire across different patrimonies is significant. This choice is substantiated by the low probability that two distinct populations chose the same characters for a same repertoire through convergence only [24, 36, 37]. Then, according to our knowledge on musical systematic and categorization, we considered that the presence of the same given character in the same repertoire from two distinct patrimonies implies that these patrimonies are more similar than two distinct patrimonies sharing the same character in two distinct repertoires. We therefore dismissed two alternative coding procedures that where either too much or too little discriminant. The first consists in coding for the presence of characters in each repertoire following a binary system. This coding system favours the hypothesis of strict resemblance, which states that to infer a common origin for two patrimonies, a given character must be present in the same repertoire of the cultural groups considered. There must therefore be strict homology between these different character states. The second, ‘soft’ coding procedure consists in coding character states without linking them to the repertoire in which they are present. This method gives preference to convergence, thereby weakening the signal from vertical transmission. Indeed, distinct musical patrimonies may use the same character because of the intrinsic limits of the musical system without hinting to any relationship between these patrimonies.

All these considerations call for a multi-state rather than a binary coding system for each character which takes into consideration the repertoires where this character can be found. Absence was coded with a zero, missing data with a question mark.

### Testing treeness

The matrix contained 58 taxa and 322 characters, among which 203 are parsimony-informative.

In oral tradition, a musical patrimony is a set of musical component transmitted vertically from one generation to the next. Therefore, musical patrimonies can be considered as a cultural artefact maintaining evolutionary signal needed for reconstructing phylogenetic history. In order to estimate tree-likeness of the musical patrimonies we both used a non-cladistic method -SPLITSTREE4 [38]- and a cladistic method: standard parsimony [39]. Note that for using SPLITSTREE4, we had to code the intrinsic characters in binary system. As NeighborNet method can overfit the data, splits with small weights (less than 0.005) were filtered from the split graph as in Gray et al. [40].

As to the cladistic method, standard parsimony approach was conducted using PAUP4.0b10 [39]. Characters were treated as unordered and unweighted in the search of the most parsimonious trees. Heuristic searches were performed with 1000 random addition sequences and TBR branch swapping. Homoplasy optimization was performed under ACCTRAN. Indeed, parsimonious trees allow to infer ancestral states at a given node, and in case of homoplasy ACCTRAN favours reversals over convergences. However, although the parsimony approach was chosen for reconstructing ancestral musical characters and studying synapomorphies and homoplasy, the precise analysis of synapomorphies is out of the scope of the present paper.

We retained tree length, Consistency Index (CI) and Retention Index (RI) as relevant criteria to discuss the structure of our data. CI = M/S, where M is the minimum number of character changes given the number of character states, and S the actual number of character changes (“steps”) required in the most parsimonious tree(s). CI may be overestimated because of autapomorphic changes (i.e., changes unique to a given terminal group). The Retention Index corrects for this. RI = (G-S)/(G-M), where G is the maximum number of steps implied by the matrix (the number of steps of a tree with a single node, i.e. no resolution at all), M is the minimum of steps implied by the matrix (the sum of character amplitudes), and S is the number of necessary steps in the most parsimonious tree(s) [35]. RI measures the excess of homoplasy regardless of autapomorphic changes.

The tree was rooted using an all-zero hypothetical ancestor. This is justified by the fact that, in the coding of character states, a “zero” symbol was given to code for the absence of a given state. The rooting methodology we chose therefore implies that we preferred to consider the appearance of a character state from its absence rather than its absence derived from presence followed by disappearance. This is a rational approach in the absence of any *a priori* data or fact to support such a reversal.

Support of the branches was calculated using a non-resampling method in order to keep the internal structure of the data, i.e. Bremer support [41]. The Bremer support for a node is simply the extra length (expressed in number of steps) needed to lose the node in the strict consensus of near-most parsimonious trees.

### Ethnomusicological coding

We collected data for 58 musical patrimonies from 23 farmer groups and 5 pygmies groups. Table 1 describes the 58 taxa included in the matrix, together with a map (Fig 1) showing the localization of the corresponding patrimonies. We identified in total 161 distinct repertoires, according to their endogenous name, context of performance and social or symbolic functions. We listed in total 63 instruments. Using direct observation of musical performance, we were able to identify two vocal techniques, the jodel [42, 43], and pseudo-jodel, which is characterized by great intervals jumps and a distinct tone but without changing laryngal configuration. We also identified three polyphonic processes, homorhythmy, counterpoint, and partial-counterpoint [44], as well as two vocal forms, antiphonal and responsorial. Musical analysis of pieces allowed us to identify 14 metric values, 64 rhythmic values (25 binary and 39 ternary), and 12 scales values. S1 Table describes the 322 characters identified for building the tree.

**Table 1:** Description of the taxons. In column 2 is a code for the map Fig 1; column 3 name of the ethnic group and its localisation; column 3: province; column 4: side of the Ogooue river.

	Code	Musical patrimony of	Province	River
Ngomsekaseka	P1	the ngom ethnic group from Sekaseka village	Ngounie	South Ogooue
Tsoghosekaseka	M2	the tsogho ethnic group from Sekaseka village.	Ngounie	South Ogooue
Ngommimongo	P3	the ngom ethnic group from Mimongo town.	Ngounie	South Ogooue
Bongoytranquille	A4	the pygmies bongo ethnic group from Tranquille village.	Ngounie	South Ogooue
Bongotchibangavillage	A5	the pygmies bongo ethnic group from Tchibanga village.	Ngounie	South Ogooue
Bongonyoye2	A6	the pygmies bongo ethnic group from Nyoye 2 village.	Ngounie	South Ogooue
Tsoghonyoye2	M7	the tsogho ethnic group from Nyoye 2 village	Ngounie	South Ogooue
Tsoghomatadi7	M8	the tsogho ethnic group from Matadi 7 village.	Ngounie	South Ogooue
Moghamademambi	M9	the pygmies moghama ethnic group from Mambi village.	Nyanga	South Ogooue
Punudemandilou	M10	the punu ethnic group from Mandilou village.	Nyanga	South Ogooue
Punudedouano2	M11	the punu ethnic group from Douano 2 village.	Nyanga	South Ogooue
Ngommouallo	P12	the akélé ethnic group from Mouallo village.	Ngounie	South Ogooue
Bongongando	P13	the pygmies bongo ethnic group from Ngando village.	Ngounie	South Ogooue
NgomBolapisa	P14	the akélé ethnic group from Bolapisa village.	Ngounie	South Ogooue
BongoMuyiku	A15	the pygmies bongo ethnic group from Muyiku village.	Ngounie	South Ogooue
ApinjidestMartin	M16	the apinji ethnic group from Saint Martin des Apinji village.	Ngounie	South Ogooue
Ngommanamana	P17	the ngom ethnic group from Manamana village.	Ogooue Lolo	South Ogooue
Bongomanamana	A18	the pygmies bongo ethnic group from Manamana village.	Ogooue Lolo	South Ogooue
Awandjlipaka1	M19	the awandji ethnic group from Lipaka 1 village.	Ogooue Lolo	South Ogooue
AwandjiDoume	M20	the awandji ethnic group from Dume village.	Ogooue Lolo	South Ogooue
BongoDoume	A21	the pygmies bongo ethnic group from Doume village.	Ogooue Lolo	South Ogooue
MurimbeMandilou	A22	the pygmies murimbe ethnic group from Mandilou village.	Nyanga	South Ogooue
GisiramandiloNgounie	M23	the gisir ethnic group from Mandilou village	Ngounie	South Ogooue
Galoa	M24	the galoa ethnic group from Moyen Ogooue province	Moyen Ogooue	South Ogooue
Orungu	M25	the orungu ethnic group from Ogooue maritime province	Ogooue Maritime	South Ogooue
Nkomi	M26	the nkomi ethnic group from Ogooue maritime province	Ogooue Maritime	South Ogooue
Teke	P27	the teke ethnic group around Franceville town.	Haut Ogooue	South Ogooue
TekedeObia1	P28	the teke ethnic group from Obia 1 village	Haut Ogooue	South Ogooue
TekedeObegue	P29	the teke ethnic group from Obegue village	Haut Ogooue	South Ogooue
ObambadeOndili	M30	the obamba ethnic group from Ondili village	Haut Ogooue	South Ogooue
ObambadeEyouga1	M31	the obamba ethnic group from Eyouga 1 village	Haut Ogooue	South Ogooue
ObambadeOmoye	M32	the obamba ethnic group from Omoye village	Haut Ogooue	South Ogooue
Bakanighi	P33	the bakanighi ethnic group from Haut Ogooue province	Haut Ogooue	South Ogooue
Eshira	M34	the eshira ethnic group from Ngounié province	Ngounie	South Ogooue
Sango	M35	the sango ethnic group from Ngounié province.	Ngounie	South Ogooue
SangodeDyanga	M36	the sango ethnic group from Dyanga village.	Ngounie	South Ogooue
Puvi	M37	the puvi ethnic group from Ngounie province.	Ngounie	South Ogooue
Simba	M38	the simba ethnic group from Ngounie province.	Ngounie	South Ogooue
Ntumu	P39	the ntumu ethnic group from Woleu Ntem province.	Woleu Ntem	North Ogooue
Mveng	P40	the mveng ethnic group from Woleu Ntem province.	Woleu Ntem	North Ogooue
Okak	P41	the okak ethnic group from Woleu Ntem province.	Woleu Ntem	North Ogooue
Fang	P42	the fang ethnic group from Woleu Ntem province.	Woleu Ntem	North Ogooue
Baka	P43	the pygmies baka ethnic group from Woleu Ntem province.	Woleu Ntem	North Ogooue
KoyaEkata	P44	the pygmies koya ethnic group from Ekata village.	Ogooue Ivindo	North Ogooue
Ngom	P45	the ngom ethnic group from Ogooue Ivindo province.	Ogooue Ivindo	North Ogooue
KoyaImbong	P46	the pygmies koya ethnic group from Imbong village.	Ogooue Ivindo	North Ogooue
Mwessa	P47	the mwessa ethnic group from Ogooue Ivindo province.	Ogooue Ivindo	North Ogooue
KotadeEgoPoma	P48	the kota ethnic group from Ego Poma village.	Ogooue Ivindo	North Ogooue
KweledeMazingo	P49	the kwele ethnic group from Mazingo village.	Ogooue Ivindo	North Ogooue
BongoEkala2	A50	the pygmies bongo ethnic group from Ekala 2 village.	Haut Ogooue	South Ogooue
BongoBoumango	A51	the pygmies bongo ethnic group from Boumango town area.	Haut Ogooue	South Ogooue
Bahumbu	P52	the bahumbu ethnic group from Haut Ogooue province.	Haut Ogooue	South Ogooue
Mbahouins	P53	the mbahouins ethnic group from Haut Ogooue province.	Haut Ogooue	South Ogooue
BongodeIwatchi	A54	the pygmies bongo ethnic group from Iwatchi village.	Ogooue Lolo	South Ogooue
BongoMoghon	A55	the pygmies bongo ethnic group from Moghombofoala village.	Ogooue Lolo	South Ogooue
BongoMakoula	A56	the pygmies bongo ethnic group from Makoula village.	Ogooue Lolo	South Ogooue
BongoMidouma	A57	the pygmies bongo ethnic group from Midouma village.	Ogooue Lolo	South Ogooue
Tsoghoestuaire	M58	the tsogho ethnic group from Estuaire province.	Estuaire	North Ogooue

## Results and Discussion

### Phylogenetic tree

**Fig 2:**
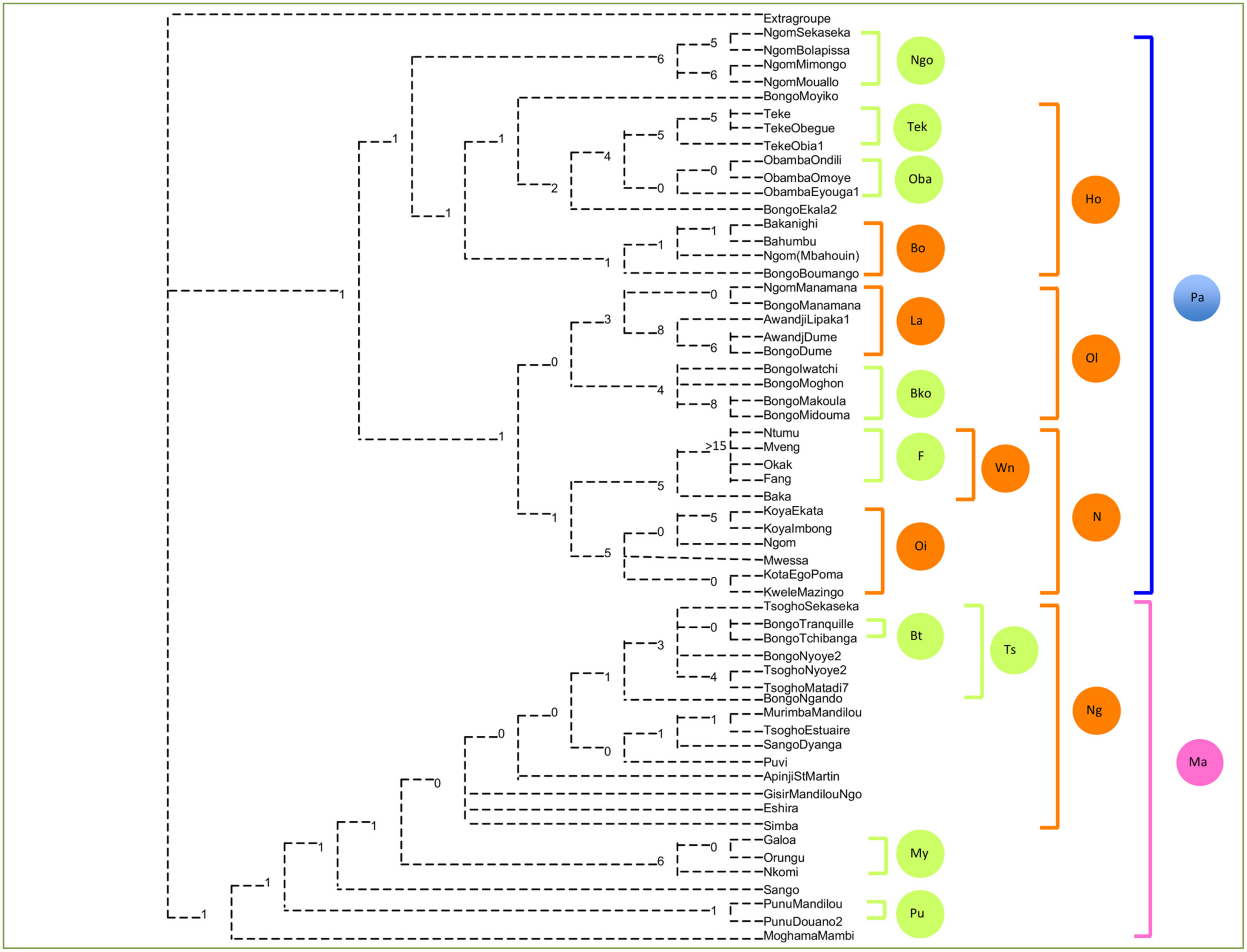
Majority-rule consensus tree of 271,160 trees of 835 steps (CI 0.6 and RI 0.7) showing relationships of musical patrimonies from Gabon. (Blue: patrilineal clade; Pink: matrilineal clade; Brown: geographic clades; Green: ethnonymic clades). Bremer indices are indicated on each node. Bremer index of 0 means that the node collapses in a strict consensus tree.

**Fig 3:**
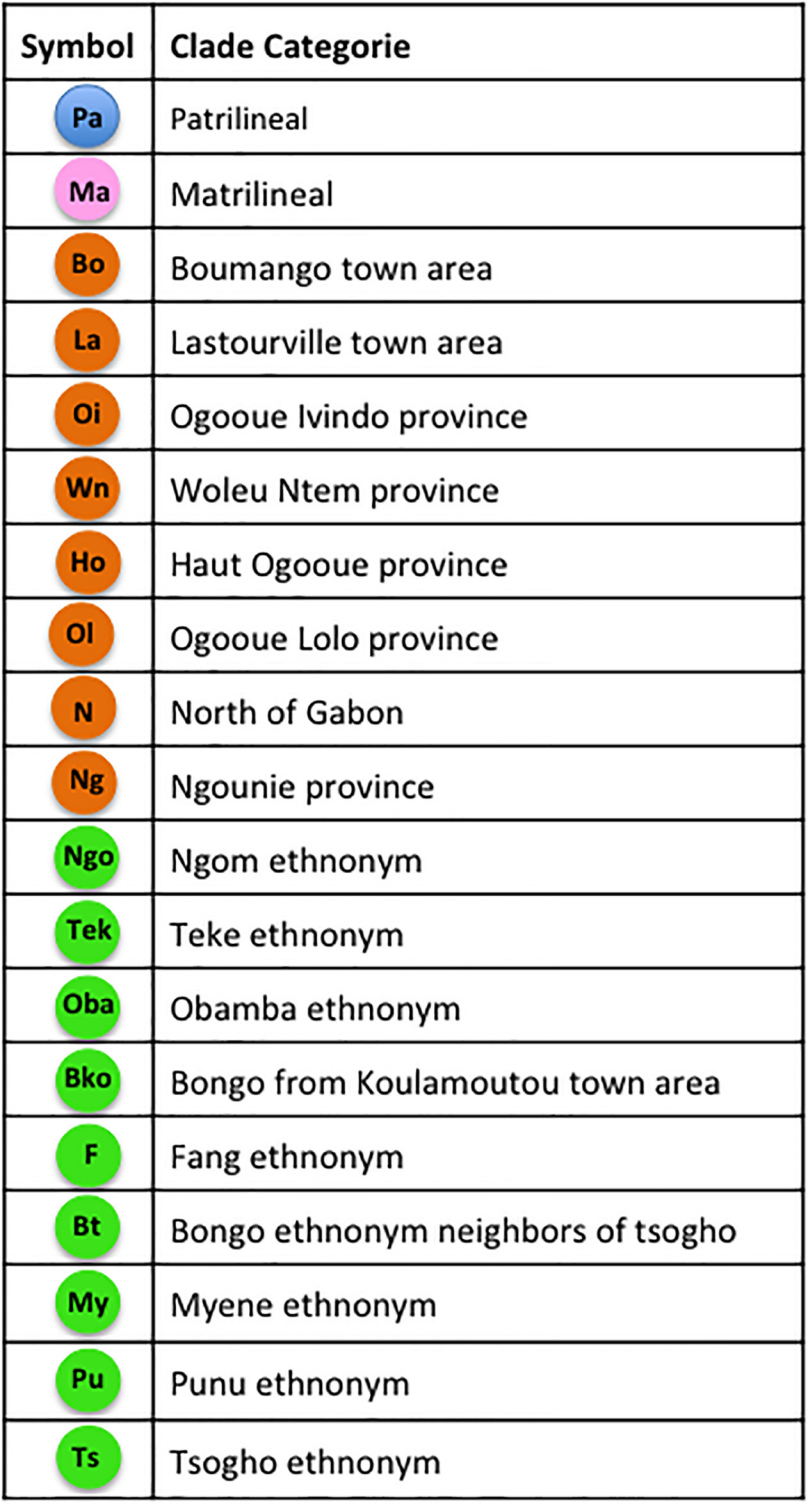
Categorization of clades. Blue and pink colours represent descent system clades. Brown colours represent geographic clades. Green colours represent ethnonymic clades.

Fig 2 shows the majority-rule consensus of 271,160 trees of 835 steps (CI = 0.6; RI = 0.7) obtained with the standard parsimony method. Fig 3 describes the clades found in Fig 2.

The tree clearly show three categories of clades: most distal clades that exhibit ethnonymic consistency (right of the tree, left column); medium nodes that are geographically consistent (right of the tree, median column); deepest nodes which reflect descent systems (right of the tree, right column). Ethnonymic clades were primarily defined by endogenous group names. Geographic clades were circumscribed by distinctive geographic units, i.e. provinces (Ho, Ol, Wn, Oi, Ng), groups of provinces (N) or towns with their network of villages accessible by road (Bo, La, Pla).

### Evidence for vertical inheritance

The high hierarchical structure of our data strongly supports vertical inheritance. Unlike in biology or linguistics, there is no evolutionary theory of musical practices. There is a diffusionist model of transmission [45], but no mechanism of transformation. Moreover, if biology clearly distinguishes between genetic inheritance and convergence, cultural data can follow multiple transmission pathways. They can be inherited from a common ancestor, borrowed through contact, or they can also disappear and be reintroduced for various contextual reasons.

**Fig 4:**
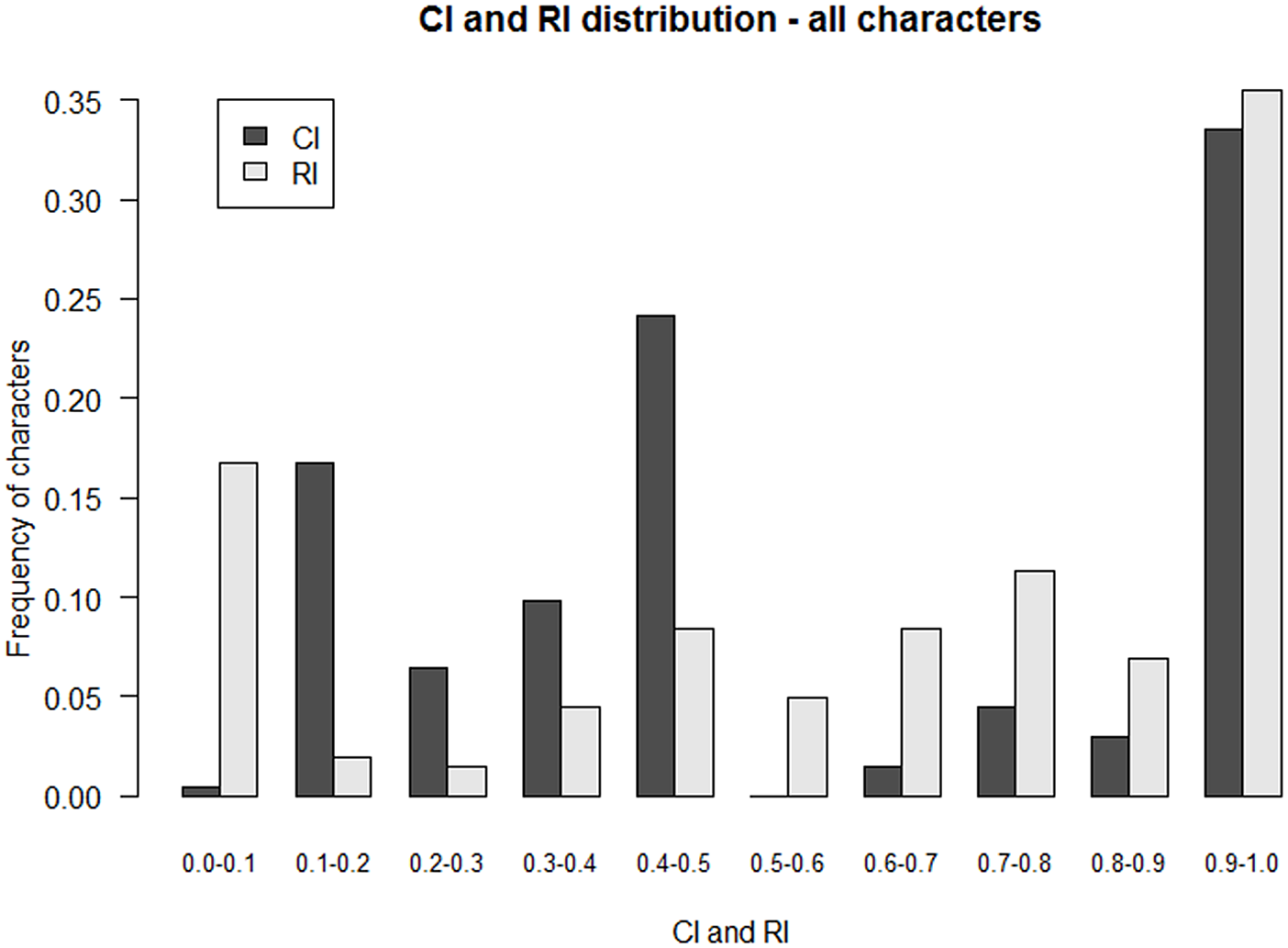
Distribution of CI and RI values across all characters. Frequencies are calculated from the number of characters informative for parsimony.

High CI and RI mean that a majority of characters seem to change in a self-consistent manner and impose their collective hierarchical structure. In other words, contradicting characters being minor, the different transmission pathways suggested above are not likely to be so diverse here. Given the size of the matrix, the values of the CI and RI (0.6 and 0.7 respectively) were remarkably high: 33,5% (68) of the characters informative for parsimony exhibited a CI of 1.0 (Fig 4).

Although parsimonious tree is first of all a hierarchical representation of character state distribution among terminal taxa and does not indicate by itself if the character states are shared because of common ancestry, or by a convergent evolution or shared by horizontal transfer (diffusion), under the diffusionist model we would have predicted an unresolved tree with a very low CI [35].

In a recent study Nunn et al, [17] simulated horizontal and vertical transmission. They show that a RI above about 0.60 is indicative of a high degree of vertically transmission and a low degree of horizontal transmission. Low C.I. and R.I. can have different explanations like diffusion and/or high rates of change, convergence, but high C.I. and R.I. are signs of vertical transmission. This is consistent with a previous study by Collard et al [46] who compared the RI of biological data with the one of cultural data. They showed that the average RI of 21 biological data was 0.61, while the average RI of the cultural data was 0.59.

The high hierarchical structure of our data therefore strongly supports vertical inheritance. This was also confirmed by a low delta-score calculated with split network methods. The non-cladistic method, SPLITSTREE4 obtained a delta score of 0.29 and a *Q*_residual score of 0.024, both indicating that the evolution of Gabonese musical patrimonies was reasonably tree-like, and therefore appropriate for phylogenetic interpretation. Those scores were similar to those obtained for by Lee [47] for the Aïnu languages study (delta = 0.25 Q-residual score = 0.01) or by Gray for Indo-European languages (delta = 0.23 Q-residual score = 0.03) [40] (see S1 Fig). These results demonstrate that the data contain a strong hierarchical consistency that is best interpreted as a signal of vertical transmission.

This result contrast with the results of Rzeszutek et al [48] who found a low evidence of treeness (delta-score = 0.46) when analyzing songs in 16 Austronesian musical patrimonies. Thus an example of vertical transmission (here in Gabon) does not exclude the possibility of strong horizontal transfers in other musical cultures somewhere else in the world, and vice-versa.

### Structure of the phylogenetic tree

We already knew that there are musical endogenous traits that define repertoires as musical categories at the population level. Our study demonstrates that some endogenous musical characters are shared and define higher categories: clades of descent rules (higher level), clades of geographic consistency (medium level), and clades of ethnonyms (smallest level). With our method, we can pinpoint what musical traits (or their association) support each clade level. All musical traits can be found in all populations, but the combination of some endogenous traits in a given set of repertoires is specific to each population and a strong identitary signal. For example, the same repertoire can be found in two populations in the same social context but with two different rhythmic formulas.

#### First level: descent rules

Interestingly, the first major branching in the tree clearly separates patrilineal (Pa) and matrilineal (Ma) populations. This is by itself a strong indication that music has something to do with human kinship systems. It is remarkable to find that descent rule is the main structuring factor of musical patrimonies since there are no obvious musical practices in direct relation to descent rule system.

In central Africa, music is highly contextualized: musical practices are strongly tied to social practices (girl or boy socialization, funerals, hunting party) and we expected to find major subdivisions that reflected these social practices. There are, conversely, no obvious musical practices in direct relation to descent rule system. It is therefore remarkable to find that descent rule is the main structuring factor of musical patrimonies.

In order to evaluate if one the three character categories (repertoire, performativ, intrinsic) could by itself determine the distinction between matrilineal and patrilineal, we did further phylogenetic analyses of each characters categories kept separated. We observed that no category alone determines clearly the matrilineal and patrilineal distinction: these two major clades, Ma and Pa, are supported by characters in the three categories.

For instance Ma and Pa are both supported by characters belonging to repertoires and in the same way they are both supported by characters belonging to musical instruments and musical scale. Conversely, rhythmics and metrics are supporting the Ma clade only. The matrilineal clade is also supported by a much larger number of musical instruments, all melodic instruments, and one instrument which is exclusively played by women. Almost all these instruments are still present today in matrilineal societies.

Regarding repertoires, some types are specific to each of the two clades: male socialisation, circumcision, fertility ceremonies, twins ceremonies, protection of resources ceremonies, and mass entertainment are traits that define the patrilineal clade. Female healing ceremonies are specific to the matrilineal clade.

Ma and Pa clades share only one type of repertoires, female socialisation, but with the absence or presence of different repertoires. In the same way, the Ma and Pa clades share one musical instrument, the drum with two membranes, but each with distinct playing techniques. While the drum is played with hands in the Ma clade, it is played with sticks in the Pa clade. Ma and Pa clades share also the hexatonic musical scale (eight notes in the octave interval) with half a tone which constitutes the core of Gabon’s musical language.

Rules of descent have a stronger impact than geography. For example the ngom populations live in the matrilineal domain but have a patrilineal rule of descent and cluster in the Pa clade. Hence, descent rules and musical patrimony co-evolve with one another as part of the same cultural ‘package’, i.e., a group of cultural elements that might co-evolve.

#### Second level: geography

At the second level of clades, populations cluster according to main geographical areas. This could reflect the geographic diffusion of musical traits. Conversely, it could also be due to common origin in a restricted area, still visible since then, as is often the case of linguistic families.

#### Third level: ethnonym

Most populations sharing the same ethnonym cluster together.

### Strong vertical inheritance of intrinsic musical characters

**Fig 5:**
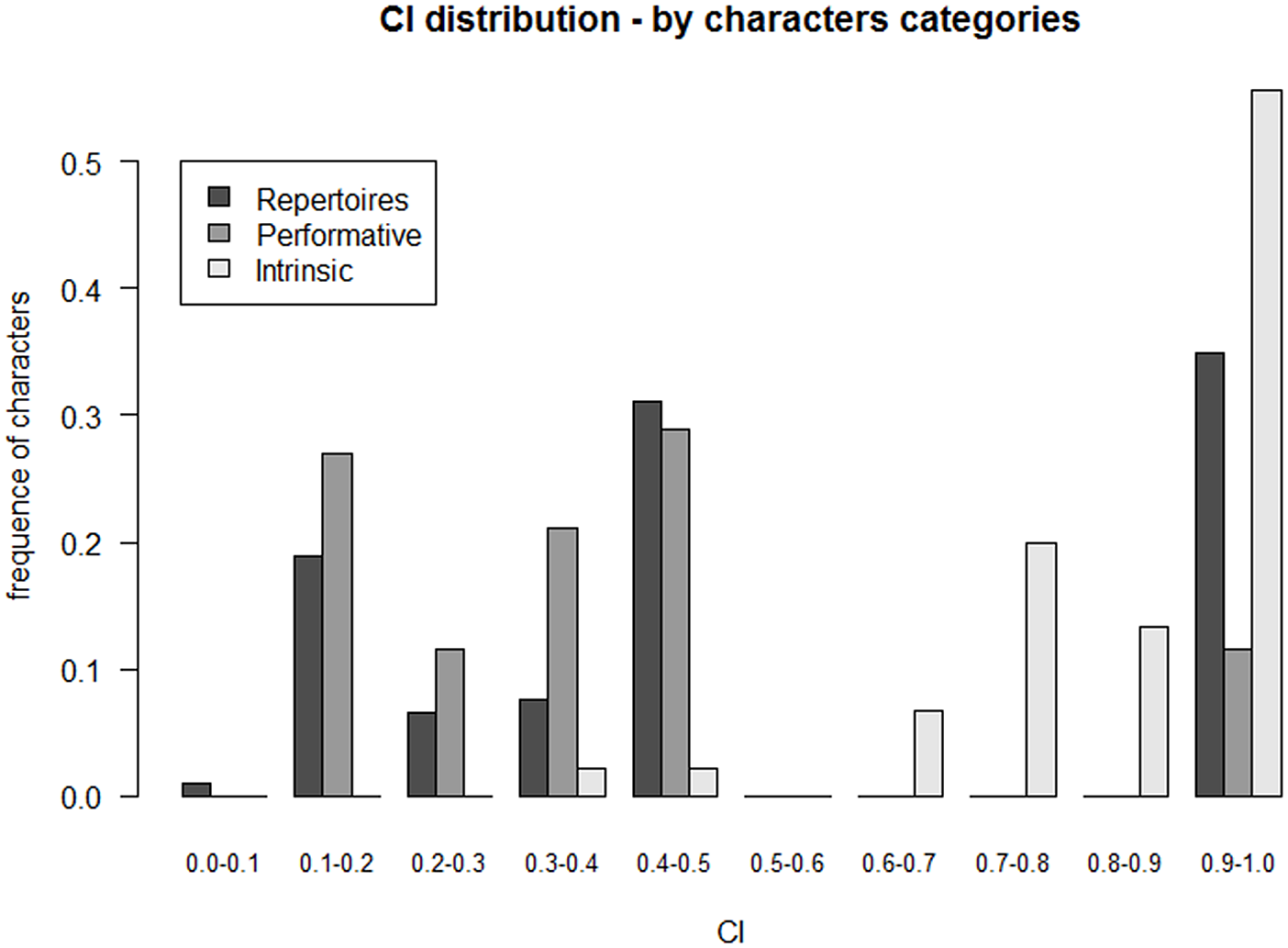
Distribution of CI values across character categories. Frequencies are calculated from the number of characters informative for parsimony of the corresponding category.

**Fig 6:**
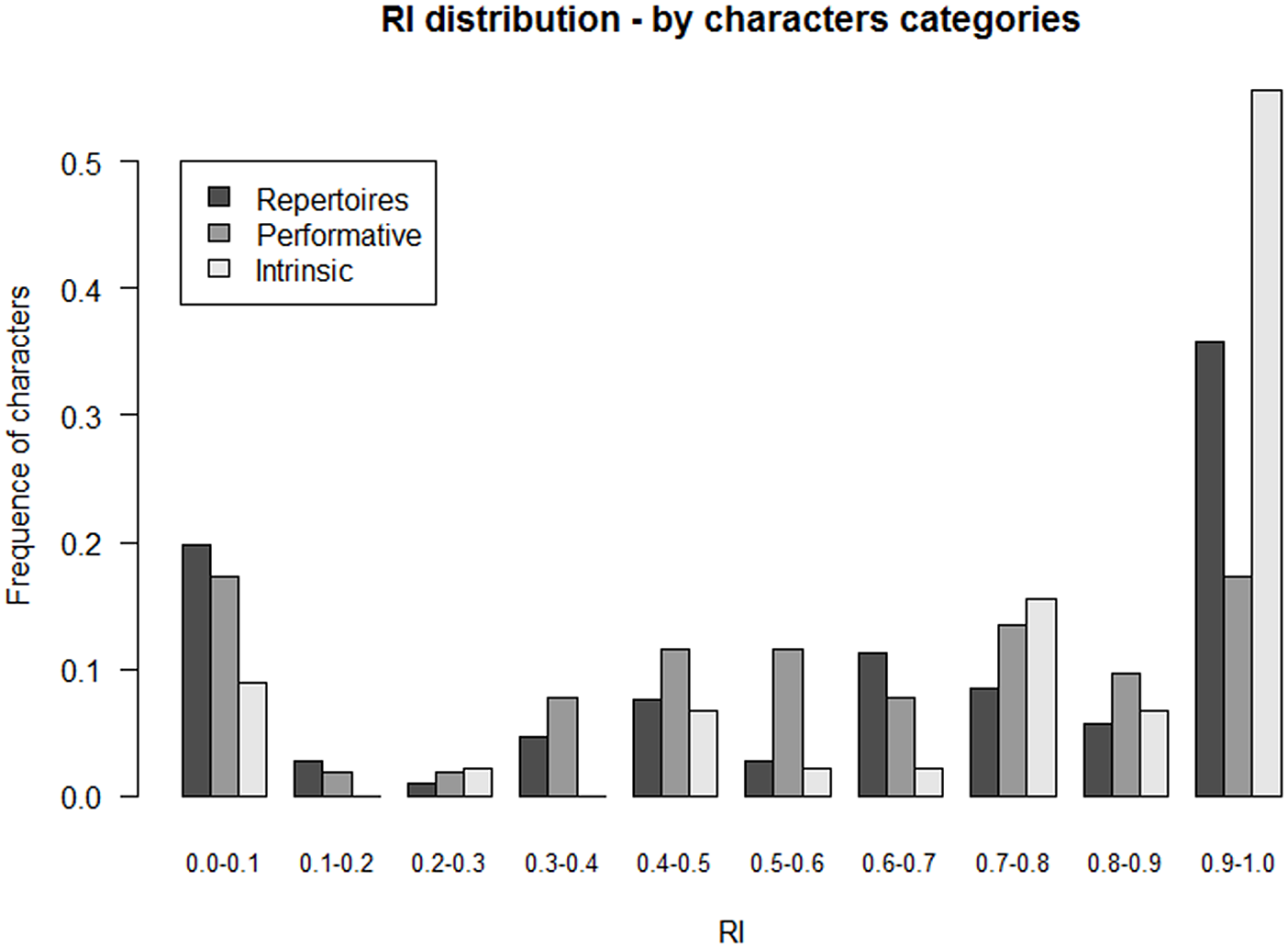
Distribution of RI values across character types. Frequencies are calculated from the number of characters informative for parsimony of the corresponding category.

A high CI means that a significant set of characters evolves in a self-consistent manner and drives the tree. Interestingly, some character categories exhibited a very high CI and RI while others did not (Figs 5 and 6).

Intrinsic characters such as rhythmic cells, metrics, and scales (45 informative characters over the total of 203) showed a strong phylogenetic signal and really formed the skeleton of the tree, with an average CI of 0.889. Repertoires, vocal techniques and forms (158 characters over the total of 203) exhibited moderate values of CI (0.408 to 0.578). Instruments (CI = 0.418) did not seem to be shared or transmitted with the same regularity as intrinsic characters. This is also true for the three homoplastic characters that encode “Polyphonic type (CI = 0.2)”. If the overall high CI associated with the tree-like hierarchy of data is to be interpreted in terms of vertical inheritance (see above), this signifies that intrinsic characters, i.e. rhythmic structures, metrics, and scale, are vertically inherited among populations and patrimonies. To a certain extent, this is also true for the repertoires, the vocal technique and forms, admitting a certain amount of homoplasy which is, also present in any data matrix under pure vertical inheritance model (e.g. in biology for molecular or anatomical data).

### The Pygmies exception

Like in any phylogenetic tree, even when descent with modification is the main explanatory model, some homoplasy results from horizontal transfers (diffusion) or convergence.

Surprisingly and against our expectations, our results do not show a clade specific to hunter-gatherer populations. A common clade for hunter-gatherer populations was initially suspected for two reasons: 1) genetic studies have shown that Pygmy populations included in the study share a recent common ancestry [27]; 2) several specific musical traits have been described in Pygmy populations, e.g. jodel and vocal polyphony using the counterpoint technique [49, 50, 51]. Our data show that Pygmy musical patrimonies are almost always closely related to that of their non-pygmy neighbors. This confirms a high level of exchange between Pygmies and non-Pygmies, which corroborates findings based on genetic and linguistic data [27, 29]. Most pygmy populations speak the same language as their non-Pygmy neighbors [29]. The fact that Pygmies easily absorb musical traits from their neighbors led to a rapid diversification of musical patrimonies among the Pygmy group. Furthermore, long term field observations have shown that changes are quite fast and that within a short period of time (2000 to 2012) Pygmy populations are able to acquire several new repertoires [26].

The multiple occurrence of hunting repertoires in the tree raises the question of convergence. Hunting repertoires are present in the majority of Pygmy and Ngom groups, but this character state cannot be explained by a hypothetical common ancestral patrimony shared by Pygmies and Ngom. Interestingly, hunting repertoires have distinct names (excepted for one repertoire shared between koya and bongo pygmies), have not the same functions, and are not performed in the same cultural contexts, as they are each related to distinct hunting techniques, all of which seems consistent with a phenomenon of convergence.

Our study further show the absence of link between subsistence and musical patrimony, in contradiction with previous studies by Lomax [52] and by Grauer [53] based on a limited diversity of hunter-gatherer populations.

## Conclusion

We found that musical data exhibit hierarchy (treeness) that is best interpreted as a strong phylogenetic signal, suggesting a predominantly vertical transmission of musical characters. Further, we show that intrinsic musical characters have the highest consistency levels and are the most likely to follow a vertical transmission pathway.

The main subdivision in musical patrimonies is the one that separates the matrilineal from patrilineal cultural domain in Gabon, emphasizing the major role of social filiation/kinship systems in structuring musical patrimonies. Conversely, there is no structuration due to the mode of subsistence (hunter gatherer *vs*. farmer). At a lower level, patrimonies are grouped according to their geographical area, thus pointing out a geographical determinant in character sharing of musical repertoires. At the third level, patrimonies are grouped according to their ethnonyms, suggesting that different ethnic groups tend to distinguish themselves from their neighbors by developing specific characters. Finally, at the lowest level, i.e. at the population level, diversification within each ethnonymic group can be explained mainly by the specific contact that each population has with its neighbors from distinct ethnonymic groups.

The fact that different populations sharing the same ethnonym cluster together indicates that ethnonyms are not merely an abstract construction by European scholars, but a tangible endogenous cultural unit.

In summary, musical innovations produced by human groups to differentiate themselves from one another are translated here through clades of ethnonymic or geographical consistencies. However these innovations remain subordinated to rules of musical grammar imposed by rules of descent, showing that music has deeper relationships with genealogy than previously thought.

We found a strong phylogenetic signal in intrinsic musical characters and demonstrated that similarities in music results from a high level of vertical cultural inheritance, thus opening new ways for studying musical evolution. Because intrinsic musical characters showed the highest level of consistency, they are the most likely to follow a vertical transmission pattern and should be the first to be considered when interest is devoted to ancestral heritage of music.

## Supporting Information

S1 Dataset Data matrix. Column 1: 58 musical patrimonies (a patrimony is a set of musical features shared by a population). They are described in the Table 1. Characters 1–161: repertoire; Characters 162–224: instrument; Characters 225–288: rhythmic cell; Characters 289–302: metric; Characters 303–315: musical scale; Characters 316–318: polyphonic processes; Characters 319–320: vocal form; Characters 321–322: vocal technique.
(XLSX)

S1 Fig A split graph showing the results of SPLITSTREE4 analyses of the Gabonese musical data. Scale bar, 0.1.
(PDF)

S1 Table Description of the 322 characters and their different states. Column 1: character number; Column 2: character name; Column 3: character type; Column 4: character states.
(PDF)
