# Supplementary figures and images for: The Evolution of Musical Diversity: The Key Role of Vertical Transmission
Source: PLoS One. 2016 Mar 30;11(3):e0151570. doi: 10.1371/journal.pone.0151570 (PMC4814106; doi:10.1371/journal.pone.0151570)

0.01

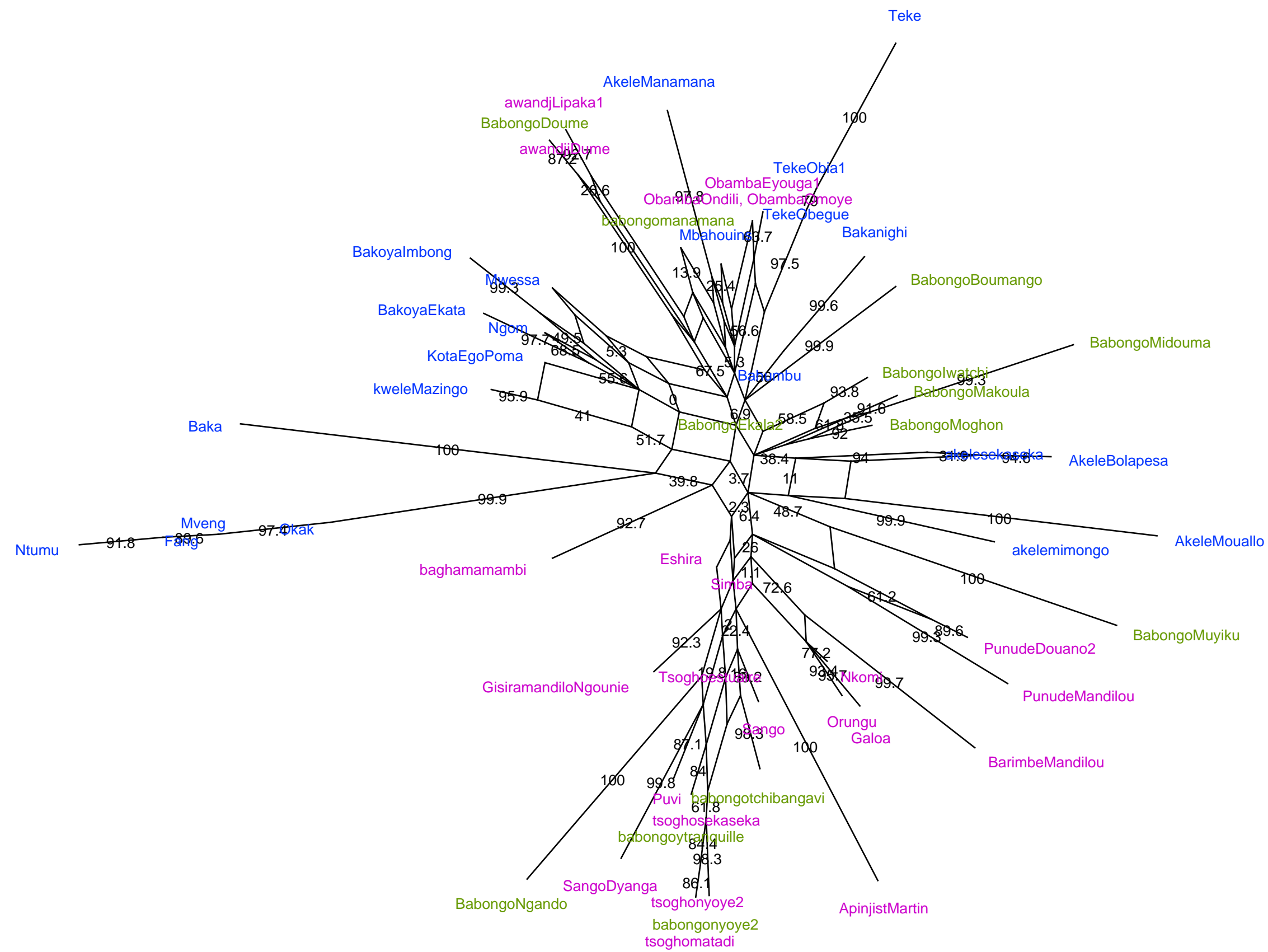

Supplement: S1 Fig — Scale bar, 0.1. (PDF) [file pone.0151570.s002.pdf]
