# Supplementary material for: The Evolution of Musical Diversity: The Key Role of Vertical Transmission
Source: PLoS One. 2016 Mar 30;11(3):e0151570. doi: 10.1371/journal.pone.0151570 (PMC4814106; doi:10.1371/journal.pone.0151570)
Supplement: S1 Table — Column 1: character number; Column 2: character name; Column 3: character type; Column 4: character states. (PDF) [file pone.0151570.s003.pdf]

1	Abale	Hunt and healing ceremonies repertoire.	Absence is coded by 0<br>Presence is coded by 1<br>Missing information is coded by ?
2	Abambo	Spirit ceremonies repertoire	Absence is coded by 0<br>Presence is coded by 1<br>Missing information is coded by ?
3	Abattage des arbres	Logging repertoire	Absence is coded by 0<br>Presence is coded by 1<br>Missing information is coded by ?
4	Abwema/Ibwema	Hunt repertoire	Absence is coded by 0<br>Presence is coded by 1<br>Missing information is coded by ?
5	Agombe Nero	Ancestor cult repertoire	Absence is coded by 0<br>Presence is coded by 1<br>Missing information is coded by ?
6	Akomamba	Entertainment repertoire	Absence is coded by 0<br>Presence is coded by 1<br>Missing information is coded by ?
7	Amboko	Entertainment repertoire	Absence is coded by 0<br>Presence is coded by 1<br>Missing information is coded by ?
8	Ampaza	Twins ceremonies repertoire	Absence is coded by 0<br>Presence is coded by 1<br>Missing information is coded by ?
9	Ampoli	Male brotherhood ceremonies repertoire	Absence is coded by 0<br>Presence is coded by 1<br>Missing information is coded by ?
10	Aringa/bodu	Entertainment repertoire	Absence is coded by 0<br>Presence is coded by 1<br>Missing information is coded by ?
11	Arc en bouche	Entertainment and tales repertoire with mouth bow	Absence is coded by 0<br>Presence is coded by 1<br>Missing information is coded by ?
12	Beka	Circumcision ceremonies repertoire	Absence is coded by 0<br>Presence is coded by 1<br>Missing information is coded by ?
13	Berceuse	Lullabies repertoire	Absence is coded by 0<br>Presence is coded by 1<br>Missing information is coded by ?
14	Bodi	Male brotherhood ceremonies repertoire	Absence is coded by 0<br>Presence is coded by 1<br>Missing information is coded by ?
15	Boluma	Hunt ceremonies repertoire	Absence is coded by 0<br>Presence is coded by 1<br>Missing information is coded by ?
16	Bubeyi	Male brotherhood ceremonies repertoire	Absence is coded by 0<br>Presence is coded by 1<br>Missing information is coded by ?
17	Ngoye	Male brotherhood ceremonies repertoires	Absence is coded by 0<br>Presence is coded by 1<br>Missing information is coded by ?
18	Buma	Male brotherhood ceremonies	Absence is coded by 0

		repertoires.	Presence is coded by 1<br>Missing information is coded by ?
19	Bumba	Divination ceremonies repertoire	Absence is coded by 0<br>Presence is coded by 1<br>Missing information is coded by ?
20	Bwiti akoa	Male brotherhood ceremonies repertoire	Absence is coded by 0<br>Presence is coded by 1<br>Missing information is coded by ?
21	Bwiti Dissumba	Male brotherhood ceremonies repertoire	Absence is coded by 0<br>Presence is coded by 1<br>Missing information is coded by ?
22	Bwiti Mitombwé	Healing ceremonies repertoire	Absence is coded by 0<br>Presence is coded by 1<br>Missing information is coded by ?
23	Bwiti Michoko	Healing ceremonies repertoire	Absence is coded by 0<br>Presence is coded by 1<br>Missing information is coded by ?
24	Bwiti Ndea	Male brotherhood ceremonies repertoire	Absence is coded by 0<br>Presence is coded by 1<br>Missing information is coded by ?
25	Bwiti Nzobi	Male brotherhood ceremonies repertoire	Absence is coded by 0<br>Presence is coded by 1<br>Missing information is coded by ?
26	Chasse	Different hunt ceremonies repertoires	Absence is coded by 0<br>Presence is coded by 1<br>Missing information is coded by ?
27	Chasse à l'éléphant	Elephant hunt ceremonies repertoires	Absence is coded by 0<br>Presence is coded by 1<br>Missing information is coded by ?
28	Contes	Tales repertoire	Absence is coded by 0<br>Presence is coded by 1<br>Missing information is coded by ?
29	Deke	Healing ceremonies repertoire	Absence is coded by 0<br>Presence is coded by 1<br>Missing information is coded by ?
30	Dibanzi	Female brotherhood ceremonies repertoire	Absence is coded by 0<br>Presence is coded by 1<br>Missing information is coded by ?
31	Djumbu	Twins ceremonies repertoire	Absence is coded by 0<br>Presence is coded by 1<br>Missing information is coded by ?
32	Diboka/Dibogha	Mourning and healing ceremonies repertoire	Absence is coded by 0<br>Presence is coded by 1<br>Missing information is coded by ?
33	Digjangu	Big chief mourning ceremonies repertoire	Absence is coded by 0<br>Presence is coded by 1<br>Missing information is coded by ?
34	Ditsatsa	Male brotherhood ceremonies repertoire	Absence is coded by 0<br>Presence is coded by 1<br>Missing information is coded by ?
35	Disesa	Male brotherhood and healing ceremonies repertoire	Absence is coded by 0<br>Presence is coded by 1<br>Missing information is coded by ?
36	Ebanighi	Male brotherhood ceremonies	Absence is coded by 0

		repertoire	Presence is coded by 1<br>Missing information is coded by ?
37	Ecrasage	Crushing peanuts repertoire	Absence is coded by 0<br>Presence is coded by 1<br>Missing information is coded by ?
38	Edzengi	Forest protection and maintaining social order ceremonies repertoire	Absence is coded by 0<br>Presence is coded by 1<br>Missing information is coded by ?
39	Ekunda	Entertainment repertoire	Absence is coded by 0<br>Presence is coded by 1<br>Missing information is coded by ?
40	Elenge	Female spirit ceremonies repertoire	Absence is coded by 0<br>Presence is coded by 1<br>Missing information is coded by ?
41	Elombo	Female healing brotherhood ceremonies repertoire	Absence is coded by 0<br>Presence is coded by 1<br>Missing information is coded by ?
42	Enangue (moburu)	Entertainment repertoire	Absence is coded by 0<br>Presence is coded by 1<br>Missing information is coded by ?
43	Ghebunzi	Male brotherhood ceremonies repertoire	Absence is coded by 0<br>Presence is coded by 1<br>Missing information is coded by ?
44	Gheyombo	Twins ceremonies repertoire	Absence is coded by 0<br>Presence is coded by 1<br>Missing information is coded by ?
45	Gjanga	Twins ceremonies repertoire	Absence is coded by 0<br>Presence is coded by 1<br>Missing information is coded by ?
46	Gol	Entertainment repertoire	Absence is coded by 0<br>Presence is coded by 1<br>Missing information is coded by ?
47	Inkinda	Circumcision ceremonies repertoire	Absence is coded by 0<br>Presence is coded by 1<br>Missing information is coded by ?
48	Inonga	Entertainment repertoire	Absence is coded by 0<br>Presence is coded by 1<br>Missing information is coded by ?
49	Ivanga	Female brotherhood ceremonies repertoire	Absence is coded by 0<br>Presence is coded by 1<br>Missing information is coded by ?
50	Kenge	Mourning ceremonies repertoire	Absence is coded by 0<br>Presence is coded by 1<br>Missing information is coded by ?
51	Kondjo	Entertainment repertoire	Absence is coded by 0<br>Presence is coded by 1<br>Missing information is coded by ?
52	Kono	Male brotherhood ceremonies repertoire	Absence is coded by 0<br>Presence is coded by 1<br>Missing information is coded by ?
53	Konzi	Healing ceremonies repertoire	Absence is coded by 0<br>Presence is coded by 1<br>Missing information is coded by ?
54	Koro	Entertainement repertoire	Absence is coded by 0

			Presence is coded by 1<br>Missing information is coded by ?
55	Kose	Entertainment repertoire	Absence is coded by 0<br>Presence is coded by 1<br>Missing information is coded by ?
56	Kudwé	Circumcision ceremonies repertoire	Absence is coded by 0<br>Presence is coded by 1<br>Missing information is coded by ?
57	Kunda2	Entertainement repertoire	Absence is coded by 0<br>Presence is coded by 1<br>Missing information is coded by ?
58	Laka	Entertainement repertoire	Absence is coded by 0<br>Presence is coded by 1<br>Missing information is coded by ?
59	Langhu	Hunt ceremonies repertoire	Absence is coded by 0<br>Presence is coded by 1<br>Missing information is coded by ?
60	Lenguégué	Entertainement repertoire	Absence is coded by 0<br>Presence is coded by 1<br>Missing information is coded by ?
61	Likobo	Children repertoire	Absence is coded by 0<br>Presence is coded by 1<br>Missing information is coded by ?
62	Lingwala	Entertainement repertoire	Absence is coded by 0<br>Presence is coded by 1<br>Missing information is coded by ?
63	Lisimbu	Female brotherhood ceremonies repertoire	Absence is coded by 0<br>Presence is coded by 1<br>Missing information is coded by ?
64	Lulu	Entertainement repertoire	Absence is coded by 0<br>Presence is coded by 1<br>Missing information is coded by ?
65	Mabandji	Female healing brotherhood ceremonies repertoire	Absence is coded by 0<br>Presence is coded by 1<br>Missing information is coded by ?
66	Mabundi	Female healing brotherhood ceremonies repertoire	Absence is coded by 0<br>Presence is coded by 1<br>Missing information is coded by ?
67	Ghevegha Maboma	Mourning ceremonies repertoire	Absence is coded by 0<br>Presence is coded by 1<br>Missing information is coded by ?
68	Madamba	Entertainement repertoire	Absence is coded by 0<br>Presence is coded by 1<br>Missing information is coded by ?
69	Madjungu	Entertainement repertoire	Absence is coded by 0<br>Presence is coded by 1<br>Missing information is coded by ?
70	Magha	Male brotherhood ceremonies repertoire	Absence is coded by 0<br>Presence is coded by 1<br>Missing information is coded by ?
71	Malamu	Female brotherhood repertoire	Absence is coded by 0<br>Presence is coded by 1<br>Missing information is coded by ?
72	Mandolo	Entertainement repertoire	Absence is coded by 0

			Presence is coded by 1<br>Missing information is coded by ?
73	Manele	Female brotherhood repertoire	Absence is coded by 0<br>Presence is coded by 1<br>Missing information is coded by ?
74	Mangadi	Healing ceremonies repertoire	Absence is coded by 0<br>Presence is coded by 1<br>Missing information is coded by ?
75	Mangelebo	Mourning ceremonies repertoire.	Absence is coded by 0<br>Presence is coded by 1<br>Missing information is coded by ?
76	Mariage	Wedding ceremonies repertoire	Absence is coded by 0<br>Presence is coded by 1<br>Missing information is coded by ?
77	Mavunda	healing ceremonies repertoire	Absence is coded by 0<br>Presence is coded by 1<br>Missing information is coded by ?
78	Mawandji	Spirit and healing ceremonies repertoire	Absence is coded by 0<br>Presence is coded by 1<br>Missing information is coded by ?
79	Mawass	Twins ceremonies repertoire	Absence is coded by 0<br>Presence is coded by 1<br>Missing information is coded by ?
80	Mbla	Entertainement repertoire	Absence is coded by 0<br>Presence is coded by 1<br>Missing information is coded by ?
81	Mbali	Entertainement repertoire	Absence is coded by 0<br>Presence is coded by 1<br>Missing information is coded by ?
82	Mbadi	Entertainement repertoire	Absence is coded by 0<br>Presence is coded by 1<br>Missing information is coded by ?
83	Mbudi	Entertainement repertoire	Absence is coded by 0<br>Presence is coded by 1<br>Missing information is coded by ?
84	Mbumba Yano	Spirit and healing ceremonies repertoire	Absence is coded by 0<br>Presence is coded by 1<br>Missing information is coded by ?
85	Mebassi	Entertainement repertoire	Absence is coded by 0<br>Presence is coded by 1<br>Missing information is coded by ?
86	Medzang	Entertainement repertoire	Absence is coded by 0<br>Presence is coded by 1<br>Missing information is coded by ?
87	Mekom/Makuma	Entertainment part of ritual ceremonies repertoire	Absence is coded by 0<br>Presence is coded by 1<br>Missing information is coded by ?
88	Mekueng	Entertainement with snail shells repertoire	Absence is coded by 0<br>Presence is coded by 1<br>Missing information is coded by ?
89	Meloka	Entertainement repertoire	Absence is coded by 0<br>Presence is coded by 1<br>Missing information is coded by ?
90	Mengan	Entertainement repertoire	Absence is coded by 0

			Presence is coded by 1<br>Missing information is coded by ?
91	Mikudu	Entertainement repertoire	Absence is coded by 0<br>Presence is coded by 1<br>Missing information is coded by ?
92	Mimbwiri	Spirit and healing ceremonies repertoire	Absence is coded by 0<br>Presence is coded by 1<br>Missing information is coded by ?
93	Minonze2	Entertainement repertoire	Absence is coded by 0<br>Presence is coded by 1<br>Missing information is coded by ?
94	Moburu	Entertainement repertoire	Absence is coded by 0<br>Presence is coded by 1<br>Missing information is coded by ?
95	Mogoulou	healing ceremonies repertoire	Absence is coded by 0<br>Presence is coded by 1<br>Missing information is coded by ?
96	Mokuya	Male brotherhood ceremonies repertoire	Absence is coded by 0<br>Presence is coded by 1<br>Missing information is coded by ?
97	Mokuyi	Male brotherhood ceremonies repertoire	Absence is coded by 0<br>Presence is coded by 1<br>Missing information is coded by ?
98	Mopfougou	Entertainement repertoire	Absence is coded by 0<br>Presence is coded by 1<br>Missing information is coded by ?
99	Mosango	Circumcision ceremonies repertoire	Absence is coded by 0<br>Presence is coded by 1<br>Missing information is coded by ?
100	Mudimu	Male brotherhood ceremonies repertoire	Absence is coded by 0<br>Presence is coded by 1<br>Missing information is coded by ?
101	Mujajo	Entertainment repertoire	Absence is coded by 0<br>Presence is coded by 1<br>Missing information is coded by ?
102	Mujishi	Mourning ceremonies repertoire	Absence is coded by 0<br>Presence is coded by 1<br>Missing information is coded by ?
103	Mukwanga	Entertainment repertoire	Absence is coded by 0<br>Presence is coded by 1<br>Missing information is coded by ?
104	Mundukwe	Hunt and male brotherhood ceremonies repertoire	Absence is coded by 0<br>Presence is coded by 1<br>Missing information is coded by ?
105	Mungala	Circumcision and twins ceremonies repertoire	Absence is coded by 0<br>Presence is coded by 1<br>Missing information is coded by ?
106	Muri (yengé)	Male brotherhood ceremonies repertoire	Absence is coded by 0<br>Presence is coded by 1<br>Missing information is coded by ?
107	Muyongo	Mourning ceremonies repertoire	Absence is coded by 0<br>Presence is coded by 1<br>Missing information is coded by ?
108	Mvet	Male withdrawal of mourning	Absence is coded by 0

		and epic entertainement repertoire	Presence is coded by 1<br>Missing information is coded by ?
109	Mvudi	Male brotherhood ceremonies repertoire	Absence is coded by 0<br>Presence is coded by 1<br>Missing information is coded by ?
110	Mwiri	Male brotherhood ceremonies repertoire	Absence is coded by 0<br>Presence is coded by 1<br>Missing information is coded by ?
111	Ndamba	entertainement repertoire	Absence is coded by 0<br>Presence is coded by 1<br>Missing information is coded by ?
112	Ndombi	Plantation labour repertoire	Absence is coded by 0<br>Presence is coded by 1<br>Missing information is coded by ?
113	Ndji (èngne)	Epic entertainement and war ceremonies repertoire	Absence is coded by 0<br>Presence is coded by 1<br>Missing information is coded by ?
114	Nganga	Healing ceremonies repertoire	Absence is coded by 0<br>Presence is coded by 1<br>Missing information is coded by ?
115	Ngi	Ancestor cult ceremonies repertoire	Absence is coded by 0<br>Presence is coded by 1<br>Missing information is coded by ?
116	Ngungutsogho (Gongo)	judge customary ceremonies repertoire	Absence is coded by 0<br>Presence is coded by 1<br>Missing information is coded by ?
117	Ngungu	Entertainement repertoire	Absence is coded by 0<br>Presence is coded by 1<br>Missing information is coded by ?
118	Ngwata	Entertainement repertoire	Absence is coded by 0<br>Presence is coded by 1<br>Missing information is coded by ?
119	Ngwala	Entertainement repertoire	Absence is coded by 0<br>Presence is coded by 1<br>Missing information is coded by ?
120	Njembé (Bo)	Female brotherhood ceremonies repertoire	Absence is coded by 0<br>Presence is coded by 1<br>Missing information is coded by ?
121	Nlup	Entertainement repertoire	Absence is coded by 0<br>Presence is coded by 1<br>Missing information is coded by ?
122	Nubeke	Repertoire	Absence is coded by 0<br>Presence is coded by 1<br>Missing information is coded by ?
123	Nyas	Male lifting of mourning ceremonies repertoire	Absence is coded by 0<br>Presence is coded by 1<br>Missing information is coded by ?
124	Nzobi	Male brotherhood ceremonies repertoire	Absence is coded by 0<br>Presence is coded by 1<br>Missing information is coded by ?
125	Nzegho (nje)	Male brotherhood ceremonies repertoire	Absence is coded by 0<br>Presence is coded by 1<br>Missing information is coded by ?
126	Okungu	Repertoire	Absence is coded by 0

			Presence is coded by 1<br>Missing information is coded by ?
127	Olamagha	Mourning repertoire	Absence is coded by 0<br>Presence is coded by 1<br>Missing information is coded by ?
128	Olende	Plantation labour repertoire	Absence is coded by 0<br>Presence is coded by 1<br>Missing information is coded by ?
129	Olobo	Hunt preparation repertoire	Absence is coded by 0<br>Presence is coded by 1<br>Missing information is coded by ?
130	Ologo	Female healing brotherhood ceremonies repertoire	Absence is coded by 0<br>Presence is coded by 1<br>Missing information is coded by ?
131	Ombosso	Entertainement repertoire	Absence is coded by 0<br>Presence is coded by 1<br>Missing information is coded by ?
132	Ombwiri	Female healing brotherhood ceremonies repertoire	Absence is coded by 0<br>Presence is coded by 1<br>Missing information is coded by ?
133	Omias	Entertainement repertoire	Absence is coded by 0<br>Presence is coded by 1<br>Missing information is coded by ?
134	Onaka	Entertainement repertoire	Absence is coded by 0<br>Presence is coded by 1<br>Missing information is coded by ?
135	Onga	Male brotherhood ceremonies repertoire	Absence is coded by 0<br>Presence is coded by 1<br>Missing information is coded by ?
136	Onkila	Twins and healing ceremonies repertoire	Absence is coded by 0<br>Presence is coded by 1<br>Missing information is coded by ?
137	Otchendze	Entertainement repertoire	Absence is coded by 0<br>Presence is coded by 1<br>Missing information is coded by ?
138	Ozila	Entertainement repertoire	Absence is coded by 0<br>Presence is coded by 1<br>Missing information is coded by ?
139	Pani	Entertainement repertoire	Absence is coded by 0<br>Presence is coded by 1<br>Missing information is coded by ?
140	Pêche barrage	Dam fishing repertoire	Absence is coded by 0<br>Presence is coded by 1<br>Missing information is coded by ?
141	Pêche poison	Fishing with poison repertoire	Absence is coded by 0<br>Presence is coded by 1<br>Missing information is coded by ?
142	Pleine Lune	Full moon repertoire	Absence is coded by 0<br>Presence is coded by 1<br>Missing information is coded by ?
143	Sanza	Sanza entertainment repertoire	Absence is coded by 0<br>Presence is coded by 1<br>Missing information is coded by ?
144	Shaka	Circumcision ceremonie	Absence is coded by 0

		repertoire	Presence is coded by 1<br>Missing information is coded by ?
145	Société	entertainment repertoire	Absence is coded by 0<br>Presence is coded by 1<br>Missing information is coded by ?
146	Tchengé	Repertoire	Absence is coded by 0<br>Presence is coded by 1<br>Missing information is coded by ?
147	Travaux dans les champs	Plantation labour repertoire	Absence is coded by 0<br>Presence is coded by 1<br>Missing information is coded by ?
148	Wanga	Mourning ceremonies repertoire	Absence is coded by 0<br>Presence is coded by 1<br>Missing information is coded by ?
149	Wose	Women repertoire	Absence is coded by 0<br>Presence is coded by 1<br>Missing information is coded by ?
150	Wuya	Female brotherhood ceremonies repertoire	Absence is coded by 0<br>Presence is coded by 1<br>Missing information is coded by ?
151	Xylo sur troncs de bananiers	Male brotherhood ceremonies repertoire	Absence is coded by 0<br>Presence is coded by 1<br>Missing information is coded by ?
152	Yanga	Twins ceremonies repertoire	Absence is coded by 0<br>Presence is coded by 1<br>Missing information is coded by ?
153	Madumajen	Entertainement repertoire	Absence is coded by 0<br>Presence is coded by 1<br>Missing information is coded by ?
154	Ikoro	Hunt ceremonies repertoire	Absence is coded by 0<br>Presence is coded by 1<br>Missing information is coded by ?
155	Issogha	Male brotherhood and twins ceremonies repertoire	Absence is coded by 0<br>Presence is coded by 1<br>Missing information is coded by ?
156	Mobondjo	Entertainement repertoire	Absence is coded by 0<br>Presence is coded by 1<br>Missing information is coded by ?
157	Ndumbwé	Male brotherhood ceremonies repertoire	Absence is coded by 0<br>Presence is coded by 1<br>Missing information is coded by ?
158	Kodé	Entertainement repertoire	Absence is coded by 0<br>Presence is coded by 1<br>Missing information is coded by ?
159	Abobo	Entertainement repertoire	Absence is coded by 0<br>Presence is coded by 1<br>Missing information is coded by ?
160	Mukudji	Entertainment repertoire	Absence is coded by 0<br>Presence is coded by 1<br>Missing information is coded by ?
161	Ikwara	Entertainment repertoire	Absence is coded by 0<br>Presence is coded by 1<br>Missing information is coded by ?
162	Eight strings harp	Chordophone musical instrument	Absence is coded by 0

			Presence is coded by 1<br>Missing information is coded by ?
163	Pluriarc	Chordophone musical instrument	Absence is coded by 0<br>Presence is coded by 1<br>Missing information is coded by ?
164	Mouth bow	Chordophone musical instrument	Absence is coded by 0<br>Presence is coded by 1<br>Missing information is coded by ?
165	Bow with two strings	Chordophone musical instrument	Absence is coded by 0<br>Presence is coded by 1<br>Missing information is coded by ?
166	Harp zither	Chordophone musical instrument	Absence is coded by 0<br>Presence is coded by 1<br>Missing information is coded by ?
167	Bow with resonator	Chordophone musical instrument	Absence is coded by 0<br>Presence is coded by 1<br>Missing information is coded by ?
168	Horn	Aerophone musical instrument	Absence is coded by 0<br>Presence is coded by 1<br>Missing information is coded by ?
169	Whistle	Aerophone musical instrument	Absence is coded by 0<br>Presence is coded by 1<br>Missing information is coded by ?
170	Papaya leafstalk whistle	Aerophone musical instrument	Absence is coded by 0<br>Presence is coded by 1<br>Missing information is coded by ?
171	Bullroarer	Aerophone musical instrument	Absence is coded by 0<br>Presence is coded by 1<br>Missing information is coded by ?
172	Whistle earth	Aerophone musical instrument	Absence is coded by 0<br>Presence is coded by 1<br>Missing information is coded by ?
173	Calebash horn	Aerophone musical instrument	Absence is coded by 0<br>Presence is coded by 1<br>Missing information is coded by ?
174	Fixed key xylophones with multiple calabashes	Idiophone musical instrument	Absence is coded by 0<br>Presence is coded by 1<br>Missing information is coded by ?
175	Log xylophones	Idiophone musical instrument	Absence is coded by 0<br>Presence is coded by 1<br>Missing information is coded by ?
176	Sanza	Idiophone musical instrument	Absence is coded by 0<br>Presence is coded by 1<br>Missing information is coded by ?
177	Short sticks banging together	Idiophone musical instrument	Absence is coded by 0<br>Presence is coded by 1<br>Missing information is coded by ?
178	Long sticks banging together	Idiophone musical instrument	Absence is coded by 0<br>Presence is coded by 1<br>Missing information is coded by ?
179	Slit drum	Idiophone musical instrument	Absence is coded by 0<br>Presence is coded by 1

			Missing information is coded by ?
180	Wooden bell	Idiophone musical instrument	Absence is coded by 0<br>Presence is coded by 1<br>Missing information is coded by ?
181	Wooden bell geminate	Idiophone musical instrument	Absence is coded by 0<br>Presence is coded by 1<br>Missing information is coded by ?
182	Metal open bell struck with a hammer held.	Idiophone musical instrument	Absence is coded by 0<br>Presence is coded by 1<br>Missing information is coded by ?
183	Metal open bells with a suspended crotal for a clapper	Idiophone musical instrument	Absence is coded by 0<br>Presence is coded by 1<br>Missing information is coded by ?
184	Double metal bell	Idiophone musical instrument	Absence is coded by 0<br>Presence is coded by 1<br>Missing information is coded by ?
185	Dog bell	Idiophone musical instrument	Absence is coded by 0<br>Presence is coded by 1<br>Missing information is coded by ?
186	Hollow-sphere form metal bell	Idiophone musical instrument	Absence is coded by 0<br>Presence is coded by 1<br>Missing information is coded by ?
187	Basket rattle	Idiophone musical instrument	Absence is coded by 0<br>Presence is coded by 1<br>Missing information is coded by ?
188	Double basket rattle	Idiophone musical instrument	Absence is coded by 0<br>Presence is coded by 1<br>Missing information is coded by ?
189	Double ball rattle	Idiophone musical instrument	Absence is coded by 0<br>Presence is coded by 1<br>Missing information is coded by ?
190	Calebash rattle	Idiophone musical instrument	Absence is coded by 0<br>Presence is coded by 1<br>Missing information is coded by ?
191	Snail shells	Idiophone musical instrument	Absence is coded by 0<br>Presence is coded by 1<br>Missing information is coded by ?
192	Seeds suspension rattles	Idiophone musical instrument	Absence is coded by 0<br>Presence is coded by 1<br>Missing information is coded by ?
193	Bean seeds rattle	Idiophone musical instrument	Absence is coded by 0<br>Presence is coded by 1<br>Missing information is coded by ?
194	Suspension rattles weared to the calves	Idiophone musical instrument	Absence is coded by 0<br>Presence is coded by 1<br>Missing information is coded by ?
195	Suspension rattles weared around the waist	Idiophone musical instrument	Absence is coded by 0<br>Presence is coded by 1<br>Missing information is coded by ?
196	Beam struck	Idiophone musical instrument	Absence is coded by 0<br>Presence is coded by 1<br>Missing information is coded by ?
197	Jingles in raffia	Idiophone musical instrument	Absence is coded by 0

	skirt		Presence is coded by 1<br>Missing information is coded by ?
198	Jingles belt	Idiophone musical instrument	Absence is coded by 0<br>Presence is coded by 1<br>Missing information is coded by ?
199	Bamboo stamping tube	Idiophone musical instrument	Absence is coded by 0<br>Presence is coded by 1<br>Missing information is coded by ?
200	Gas bottle	Idiophone musical instrument	Absence is coded by 0<br>Presence is coded by 1<br>Missing information is coded by ?
201	Mirliton	Membranophone musical instrument	Absence is coded by 0<br>Presence is coded by 1<br>Missing information is coded by ?
202	Goblet drum with bound skin	Membranophone musical instrument	Absence is coded by 0<br>Presence is coded by 1<br>Missing information is coded by ?
203	Cylindrical drum with skin nailed	Membranophone musical instrument	Absence is coded by 0<br>Presence is coded by 1<br>Missing information is coded by ?
204	Short cylindrical drum	Membranophone musical instrument	Absence is coded by 0<br>Presence is coded by 1<br>Missing information is coded by ?
205	Short goblet drum with skin nailed (mokiki)	Membranophone musical instrument	Absence is coded by 0<br>Presence is coded by 1<br>Missing information is coded by ?
206	Conical drum with two laced skins, hand played	Membranophone musical instrument	Absence is coded by 0<br>Presence is coded by 1<br>Missing information is coded by ?
207	Conical drum with two laced skins, one hand and one stick played	Membranophone musical instrument	Absence is coded by 0<br>Presence is coded by 1<br>Missing information is coded by ?
208	Conical drum with two laced skins, stick played	Membranophone musical instrument	Absence is coded by 0<br>Presence is coded by 1<br>Missing information is coded by ?
209	Conical drum with one laced skin	Membranophone musical instrument	Absence is coded by 0<br>Presence is coded by 1<br>Missing information is coded by ?
210	Cylindrical drum with one skin pegged	Membranophone musical instrument	Absence is coded by 0<br>Presence is coded by 1<br>Missing information is coded by ?
211	Short cylindrical drum with one skin pegged	Membranophone musical instrument	Absence is coded by 0<br>Presence is coded by 1<br>Missing information is coded by ?
212	Cylindrical drum with one skin laced and pegged	Membranophone musical instrument	Absence is coded by 0<br>Presence is coded by 1<br>Missing information is coded by ?
213	Cylindircal drum with one skin strucked by a clump of grass	Membranophone musical instrument	Absence is coded by 0<br>Presence is coded by 1<br>Missing information is coded by ?
214	Friction drum	Membranophone musical	Absence is coded by 0

		instrument	Presence is coded by 1<br>Missing information is coded by ?
215	Six strings harp with a base.	Cordophone musical instrument	Absence is coded by 0<br>Presence is coded by 1<br>Missing information is coded by ?
216	Iron whistle	Aerophone musical instrument	Absence is coded by 0<br>Presence is coded by 1<br>Missing information is coded by ?
217	Wood whistle	Aerophone musical instrument	Absence is coded by 0<br>Presence is coded by 1<br>Missing information is coded by ?
218	Ground bow	Cordophone musical instrument	Absence is coded by 0<br>Presence is coded by 1<br>Missing information is coded by ?
219	Accordion	Aerophone musical instrument	Absence is coded by 0<br>Presence is coded by 1<br>Missing information is coded by ?
220	Drumstick stamping	Idiophone musical instrument	Absence is coded by 0<br>Presence is coded by 1<br>Missing information is coded by ?
221	Bamboo whistle (dibeka)	Aerophone musical instrument	Absence is coded by 0<br>Presence is coded by 1<br>Missing information is coded by ?
222	Clashed horns	Idiophone musical instrument	Absence is coded by 0<br>Presence is coded by 1<br>Missing information is coded by ?
223	Seeds clashed	Idiophone musical instrument	Absence is coded by 0<br>Presence is coded by 1<br>Missing information is coded by ?
224	Frame drum in chair	Membranophone musical instrument	Absence is coded by 0<br>Presence is coded by 1<br>Missing information is coded by ?
225	1	♩.	Presence in ngodja repertoire is coded by à<br>Presence in lisimbu repertoire is coded by l<br>Presence in nzobi repertoire is coded by 2<br>Presence in ngoye repertoire is coded by 5<br>Presence in mungala repertoire is coded by 6<br>Presence in wuya repertoire is coded by 8<br>Absence is coded by 0<br>Missing information is coded by ?
226	2	♩ ♪	Presence in nzokou repertoire is coded by £<br>Presence in ngodja repertoire is coded by à<br>Presence in bubeyu repertoire is coded by c<br>Presence in bwiti koji repertoire is coded by è<br>Presence in onkila repertoire is coded by h<br>Presence in mavasa repertoire is coded by l<br>Presence in ibwema repertoire is coded by o<br>Presence in ngi repertoire is coded by ù<br>Presence in mabandji repertoire is coded by x<br>Presence in lisimbu repertoire is coded by 1<br>Presence in nzobi repertoire is coded by 2<br>Presence in ngwata repertoire is coded by 3<br>Presence in ngoye repertoire is coded by 5<br>Presence in mungala repertoire is coded by 6

			Presence in wuya repertoire is coded by 8<br>Presence in mimbwiri repertoire is coded by 9<br>Absence is coded by 0<br>Missing information is coded by ?
227	3	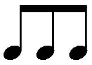	Presence in boumba repertoire is coded by $<br>Presence in nzokou repertoire is coded by £<br>Presence in ologo repertoire is coded by a<br>Presence in ngodja repertoire is coded by à<br>Presence in bubeyu repertoire is coded by c<br>Presence in langu repertoire is coded by ç<br>Presence in bwiti koji repertoire is coded by è<br>Presence in onkila repertoire is coded by h<br>Presence in ngungu tek repertoire is coded by i<br>Presence in mavasa repertoire is coded by l<br>Presence in ibwema repertoire is coded by o<br>Presence in ngi repertoire is coded by ù<br>Presence in mabandji repertoire is coded by x<br>Presence in lisimbu repertoire is coded by 1<br>Presence in nzobi repertoire is coded by 2<br>Presence in ngwata repertoire is coded by 3<br>Presence in ngoye repertoire is coded by 5<br>Presence in mungala repertoire is coded by 6<br>Presence in mopfougou repertoire is coded by 7<br>Presence in wuya repertoire is coded by 8<br>Presence in mimbwiri repertoire is coded by 9<br>Absence is coded by 0<br>Missing information is coded by ?
228	4	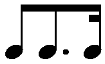	Presence in boumba repertoire is coded by $<br>Presence in nzokou repertoire is coded by £<br>Presence in ngodja repertoire is coded by à<br>Presence in bubeyu repertoire is coded by c<br>Presence in deke repertoire is coded by é<br>Presence in bwiti koji repertoire is coded by è<br>Presence in ngungu tek repertoire is coded by i<br>Presence in mavasa repertoire is coded by l<br>Presence in ibwema repertoire is coded by o<br>Presence in lisimbu repertoire is coded by 1<br>Presence in ngoye repertoire is coded by 5<br>Presence in *mungala* repertoire is coded by 6<br>Presence in *wuya* repertoire is coded by 8<br>Presence in *mimbwiri* repertoire is coded by 9<br>Absence is coded by 0<br>Missing information is coded by ?
229	5	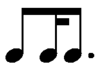	Presence in *lisimbu* repertoire is coded by 1<br>Presence in *mungala* repertoire is coded by 6<br>Absence is coded by 0<br>Missing information is coded by ?
230	6	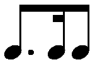	Presence in *lisimbu* repertoire is coded by 1<br>Presence in *nzobi* repertoire is coded by 2<br>Presence in *ngoye* repertoire is coded by 5<br>Presence in *mungala* repertoire is coded by 6<br>Presence in *mimbwiri* repertoire is coded by 9<br>Presence in *ngodja* repertoire is coded by à<br>Presence in *bubeyu* repertoire is coded by c

			Presence in *mavasa* repertoire is coded by l<br>Absence is coded by 0<br>Missing information is coded by ?
231	7	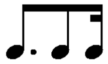	Presence in *bubeyu* repertoire is coded by c<br>Presence in *lisimbu* repertoire is coded by 1<br>Presence in *mungala* repertoire is coded by 6<br>Presence in *mimbwiri* repertoire is coded by 9<br>Absence is coded by 0<br>Missing information is coded by ?
232	8	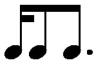	Presence in *bubeyu* repertoire is coded by c<br>Presence in *lisimbu* repertoire is coded by 1<br>Presence in *mungala* repertoire is coded by 6<br>Absence is coded by 0<br>Missing information is coded by ?
233	9	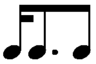	Presence in *bubeyu* repertoire is coded by c<br>Presence in *ngi* repertoire is coded by ù<br>Presence in *lisimbu* repertoire is coded by 1<br>Presence in *mungala* repertoire is coded by 6<br>Absence is coded by 0<br>Missing information is coded by ?
234	10	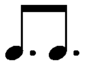	Presence in *boumba* repertoire is coded by $<br>Presence in *nzokou* repertoire is coded by £<br>Presence in *ologo* repertoire is coded by a<br>Presence in *ngodja* repertoire is coded by à<br>Presence in *bubeyu* repertoire is coded by c<br>Presence in *ibwema* repertoire is coded by o<br>Presence in *mabandji* repertoire is coded by x<br>Presence in *lisimbu* repertoire is coded by 1<br>Presence in *ngoye* repertoire is coded by 5<br>Presence in *mungala* repertoire is coded by 6<br>Presence in *wuya* repertoire is coded by 8<br>Presence in *mimbwiri* repertoire is coded by 9<br>Absence is coded by 0<br>Missing information is coded by ?
235	11	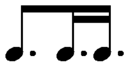	Presence in *bubeyu* repertoire is coded by c<br>Presence in *lisimbu* repertoire is coded by 1<br>Presence in *mungala* repertoire is coded by 6<br>Absence is coded by 0<br>Missing information is coded by ?
236	12	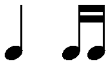	Presence in *bwiti koji* repertoire is coded by è<br>Presence in *mavasa* repertoire is coded by l<br>Presence in *ibwema* repertoire is coded by o<br>Presence in *lisimbu* repertoire is coded by 1<br>Absence is coded by 0<br>Missing information is coded by ?
237	13	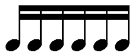	Presence in *mwiri* repertoire is coded by p<br>Presence in *mavasa* repertoire is coded by l<br>Presence in *ngoye* repertoire is coded by 5<br>Absence is coded by 0<br>Missing information is coded by ?
238	14	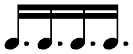	Presence in *ngoye* repertoire is coded by 5<br>Presence in *mimbwiri* repertoire is coded by 9<br>Absence is coded by 0<br>Missing information is coded by ?

239	15	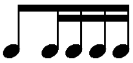	bsence is coded by 0<br>Missing information is coded by ?
240	16	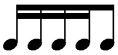	Presence in *lisimbu* repertoire is coded by 1<br>Absence is coded by 0<br>Missing information is coded by ?
241	17	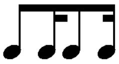	Presence in *lisimbu* repertoire is coded by 1<br>Presence in *nzobi* repertoire is coded by 2<br>Presence in *mimbwiri* repertoire is coded by 9<br>Absence is coded by 0<br>Missing information is coded by ?
242	18	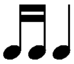	Presence in *lisimbu* repertoire is coded by 1<br>Presence in *ngoye* repertoire is coded by 5<br>Absence is coded by 0<br>Missing information is coded by ?
243	19	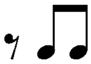	Presence in *boumba* repertoire is coded by $<br>Presence in *ngodja* repertoire is coded by à<br>Presence in *langu* repertoire is coded by ç<br>Presence in *deke* repertoire is coded by é<br>Presence in *bwiti koji* repertoire is coded by è<br>Presence in *ngungu tek* repertoire is coded by i<br>Presence in *mavasa* repertoire is coded by l<br>Presence in *ngi* repertoire is coded by ù<br>Presence in *lisimbu* repertoire is coded by 1<br>Presence in *nzobi* repertoire is coded by 2<br>Presence in *ngwata* repertoire is coded by 3<br>Presence in *ngoye* repertoire is coded by 5<br>Presence in *mungala* repertoire is coded by 6<br>Absence is coded by 0<br>Missing information is coded by ?
244	20	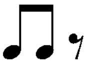	Presence in *nzobi* repertoire is coded by 2<br>Absence is coded by 0<br>Missing information is coded by ?
245	21	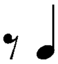	Presence in *bwiti koji* repertoire is coded by è<br>Presence in *nzobi* repertoire is coded by 2<br>Presence in *ngoye* repertoire is coded by 5<br>Presence in *mungala* repertoire is coded by 6<br>Presence in *mopfougou* repertoire is coded by 7<br>Absence is coded by 0<br>Missing information is coded by ?
246	22	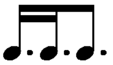	Presence in *bubeyu* repertoire is coded by c<br>Presence in *lisimbu* repertoire is coded by 1<br>Presence in *mungala* repertoire is coded by 6<br>Absence is coded by 0<br>Missing information is coded by ?
247	23	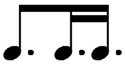	Presence in *boumba* repertoire is coded by $<br>Presence in *nzokou* repertoire is coded by £<br>Presence in *bubeyu* repertoire is coded by c<br>Presence in *ibwema* repertoire is coded by o<br>Presence in *lisimbu* repertoire is coded by 1<br>Presence in *mungala* repertoire is coded by 6<br>Presence in *wuya* repertoire is coded by 8<br>Absence is coded by 0<br>Missing information is coded by ?

248	24	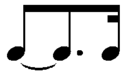	Presence in *mungala* repertoire is coded by 6<br>Absence is coded by 0<br>Missing information is coded by ?
249	25	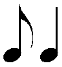	Presence in *onkila* repertoire is coded by h<br>Presence in *mbudi* repertoire is coded by l<br>Presence in *ibwema* repertoire is coded by o<br>Presence in *mwiri* repertoire is coded by p<br>Presence in *lisimbu* repertoire is coded by 1<br>Presence in *nzobi* repertoire is coded by 2<br>Presence in *ngwata* repertoire is coded by 3<br>Presence in *ngoye* repertoire is coded by 5<br>Presence in *mungala* repertoire is coded by 6<br>Presence in *mopfougou* repertoire is coded by 7<br>Presence in *wuya* repertoire is coded by 8<br>Absence is coded by 0<br>Missing information is coded by ?
250	26	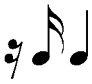	Presence in *nzobi* repertoire is coded by 2<br>0 Absence is coded by 0<br>Missing information is coded by ?
251	27	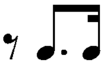	Presence in *deke* repertoire is coded by é<br>Presence in *bwiti koji* repertoire is coded by è<br>Presence in *lisimbu* repertoire is coded by 1<br>Presence in *ngoye* repertoire is coded by 5<br>Absence is coded by 0<br>Missing information is coded by ?
252	28	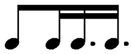	Presence in *lisimbu* repertoire is coded by 1<br>Absence is coded by 0<br>Missing information is coded by ?
253	29	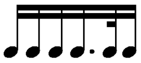	Presence in *mungala* repertoire is coded by 6<br>Absence is coded by 0<br>Missing information is coded by ?
254	30	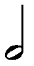	Presence in *ologo* repertoire is coded by a<br>Presence in *mbudi* repertoire is coded by f<br>Presence in *olamagha* repertoire is coded by j<br>Presence in *kenge* repertoire is coded by n<br>Presence in *makouma* repertoire is coded by s<br>Presence in *mundukwe* repertoire is coded by t<br>Presence in *kunde* repertoire is coded by v<br>Presence in *mabundi* repertoire is coded by w<br>Presence in *minonze* repertoire is coded by y<br>Presence in *lisimbu* repertoire is coded by 1<br>Presence in *mungala* repertoire is coded by 6<br>Absence is coded by 0<br>Missing information is coded by ?
255	31	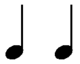	Presence in *bwiti missoko* repertoire is coded by<br>*<br>Presence in *nyembe* repertoire is coded by #<br>Presence in *ologo* repertoire is coded by a<br>Presence in *mbumba* repertoire is coded by b<br>Presence in *mbudi* repertoire is coded by f<br>Presence in *issogha* repertoire is coded by g<br>Presence in *abire nkele* repertoire is coded by k<br>Presence in *mujaja* repertoire is coded by m<br>Presence in *makouma* repertoire is coded by s

			Presence in *mundukwe* repertoire is coded by t<br>Presence in *kore* repertoire is coded by u<br>Presence in *kunde* repertoire is coded by v<br>Presence in *mabundi* repertoire is coded by w<br>Presence in *mabandji* repertoire is coded by x<br>Presence in *minonze* repertoire is coded by y<br>Presence in *bwiti disumba* repertoire is coded by z<br>Presence in *lisimbu* repertoire is coded by 1<br>Presence in *ngwata* repertoire is coded by 3<br>Presence in *mungala* repertoire is coded by 6<br>Presence in *mimbwiri* repertoire is coded by 9<br>Absence is coded by 0<br>Missing information is coded by ?
256	32	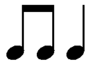	Presence in *bwiti missoko* repertoire is coded by *<br>Presence in *nyembe* repertoire is coded by #<br>Presence in *mbumba* repertoire is coded by b<br>Presence in *dijangu* repertoire is coded by r<br>Presence in *mabundi* repertoire is coded by w<br>Presence in *minonze* repertoire is coded by y<br>Absence is coded by 0<br>Missing information is coded by ?
257	33	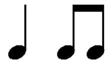	Presence in *bwiti missoko* repertoire is coded by *<br>Presence in *nyembe* repertoire is coded by #<br>Presence in *mbumba* repertoire is coded by b<br>Presence in *issogha* repertoire is coded by g<br>Presence in *olamagha* repertoire is coded by j<br>Presence in *kenge* repertoire is coded by n<br>Presence in *mujisi* repertoire is coded by q<br>Presence in *dijangu* repertoire is coded by r<br>Presence in *makouma* repertoire is coded by s<br>Presence in *mundukwe* repertoire is coded by t<br>Presence in *kore* repertoire is coded by u<br>Presence in *mabundi* repertoire is coded by w<br>Presence in *minonze* repertoire is coded by y<br>Presence in *bwiti disumba* repertoire is coded by z<br>Presence in *ngoye* repertoire is coded by 5<br>Presence in *mungala* repertoire is coded by 6<br>Absence is coded by 0<br>Missing information is coded by ?
258	34	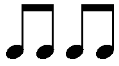	Presence in *bwiti missoko* repertoire is coded by *<br>Presence in *nyembe* repertoire is coded by #<br>Presence in *mbumba* repertoire is coded by b<br>Presence in *doka* repertoire is coded by d<br>Presence in *mbadi* repertoire is coded by e<br>Presence in *mbudi* repertoire is coded by f<br>Presence in *olamagha* repertoire is coded by j<br>Presence in *mujaja* repertoire is coded by m<br>Presence in *kenge* repertoire is coded by n<br>Presence in *makouma* repertoire is coded by s

			Presence in *mabundi* repertoire is coded by w<br>Presence in *minonze* repertoire is coded by y<br>Presence in *bwiti disumba* repertoire is coded by z<br>Presence in *lisimbu* repertoire is coded by 1<br>Presence in *ngwata* repertoire is coded by 3<br>Presence in *laka* repertoire is coded by 4<br>Presence in *ngoye* repertoire is coded by 5<br>Presence in *mungala* repertoire is coded by 6<br>Absence is coded by 0<br>Missing information is coded by ?
259	35	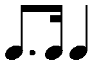	Presence in *mundukwe* repertoire is coded by t<br>Presence in *ngoye* repertoire is coded by 5<br>Absence is coded by 0<br>Missing information is coded by ?
260	36	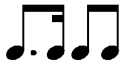	Presence in *ologo* repertoire is coded by a<br>Presence in *ngwata* repertoire is coded by 3<br>Absence is coded by 0<br>Missing information is coded by ?
261	37	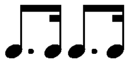	Presence in *ngoye* repertoire is coded by 5<br>Absence is coded by 0<br>Missing information is coded by ?
262	38	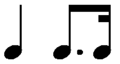	Presence in *kore* repertoire is coded by u<br>Presence in *mungala* repertoire is coded by 6<br>Absence is coded by 0<br>Missing information is coded by ?
263	41	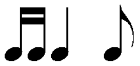	Presence in *laka* repertoire is coded by 4<br>Absence is coded by 0<br>Missing information is coded by ?
264	42	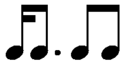	Presence in *laka* repertoire is coded by 4<br>Absence is coded by 0<br>Missing information is coded by ?
265	43	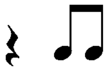	Presence in *makouma* repertoire is coded by s<br>Presence in *ngoye* repertoire is coded by 5<br>Absence is coded by 0<br>Missing information is coded by ?
266	44	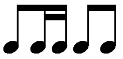	Presence in *laka* repertoire is coded by 4<br>Absence is coded by 0<br>Missing information is coded by ?
267	45	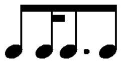	Presence in *laka* repertoire is coded by 4<br>Absence is coded by 0<br>Missing information is coded by ?
268	46	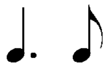	Presence in *ologo* repertoire is coded by a<br>Presence in *mbumba* repertoire is coded by b<br>Presence in *doka* repertoire is coded by d<br>Presence in *mbadi* repertoire is coded by e<br>Presence in *mbudi* repertoire is coded by f<br>Presence in *issogha* repertoire is coded by g<br>Presence in *abire nkele* repertoire is coded by k<br>Presence in *mujaja* repertoire is coded by m<br>Presence in *kenge* repertoire is coded by n<br>Presence in *dijangu* repertoire is coded by r<br>Presence in *makouma* repertoire is coded by s<br>Presence in *mundukwe* repertoire is coded by t

			Presence in *kunde* repertoire is coded by v<br>Presence in *minonze* repertoire is coded by y<br>Presence in *bwiti disumba* repertoire is coded by z<br>Presence in *lisimbu* repertoire is coded by 1<br>Presence in *mungala* repertoire is coded by 6<br>Absence is coded by 0<br>Missing information is coded by ?
269	47	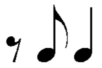	Presence in *bwiti missoko* repertoire is coded by *<br>Presence in *nyembe* repertoire is coded by #<br>Presence in *mbumba* repertoire is coded by b<br>Presence in *minonze* repertoire is coded by y<br>Presence in *mungala* repertoire is coded by 6<br>Absence is coded by 0<br>Missing information is coded by ?
270	48	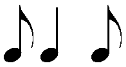	Presence in *bwiti missoko* repertoire is coded by *<br>Presence in *nyembe* repertoire is coded by #<br>Presence in *mbumba* repertoire is coded by b<br>Presence in *mbudi* repertoire is coded by f<br>Presence in *makouma* repertoire is coded by s<br>Presence in *mundukwe* repertoire is coded by t<br>Presence in *kore* repertoire is coded by u<br>Presence in *bwiti disumba* repertoire is coded by z<br>Presence in *mungala* repertoire is coded by 6<br>Absence is coded by 0<br>Missing information is coded by ?
271	49	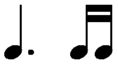	Presence in *mbudi* repertoire is coded by f<br>Absence is coded by 0<br>Missing information is coded by ?
272	50	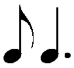	Presence in *abire nkele* repertoire is coded by k<br>Presence in *mundukwe* repertoire is coded by t<br>Presence in *kore* repertoire is coded by u<br>Presence in *mabandji* repertoire is coded by x<br>Presence in *minonze* repertoire is coded by y<br>Presence in *lisimbu* repertoire is coded by 1<br>Presence in *mungala* repertoire is coded by 6<br>Presence in *mimbwiri* repertoire is coded by 9<br>Absence is coded by 0<br>Missing information is coded by ?
273	51	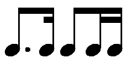	Presence in *mungala* repertoire is coded by 6<br>Absence is coded by 0<br>Missing information is coded by ?
274	52	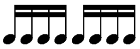	Presence in *mungala* repertoire is coded by 6<br>Absence is coded by 0<br>Missing information is coded by ?
275	53	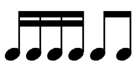	Presence in *mungala* repertoire is coded by 6<br>Absence is coded by 0<br>Missing information is coded by ?
276	54	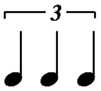	Presence in *dijangu* repertoire is coded by r<br>Presence in *makouma* repertoire is coded by s<br>Presence in *mundukwe* repertoire is coded by t

			Presence in *kore* repertoire is coded by u<br>Presence in *mimbwiri* repertoire is coded by 9<br>Absence is coded by 0<br>Missing information is coded by ?
277	55	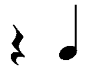	Presence in *mbumba* repertoire is coded by b<br>Presence in *mabandji* repertoire is coded by x<br>Absence is coded by 0<br>Missing information is coded by ?
278	56	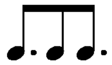	Presence in *mimbwiri* repertoire is coded by 9<br>Absence is coded by 0<br>Missing information is coded by ?
279	60	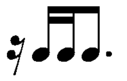	Presence in *bubeyu* repertoire is coded by c<br>Absence is coded by 0<br>Missing information is coded by ?
280	61	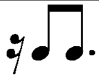	Presence in *bubeyu* repertoire is coded by c<br>Absence is coded by 0<br>Missing information is coded by ?
281	62	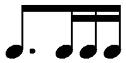	Presence in *ngoye* repertoire is coded by 5<br>Absence is coded by 0<br>Missing information is coded by ?
282	63	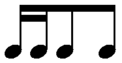	Presence in *mwiri* repertoire is coded by p<br>Presence in *ngi* repertoire is coded by ù<br>Presence in *nzobi* repertoire is coded by 2<br>Absence is coded by 0<br>Missing information is coded by ?
283	64	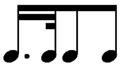	Presence in *nzobi* repertoire is coded by 2<br>Absence is coded by 0<br>Missing information is coded by ?
284	65	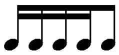	Presence in *bubeyu* repertoire is coded by c<br>Presence in *lisimbu* repertoire is coded by l<br>Absence is coded by 0<br>Missing information is coded by ?
285	66	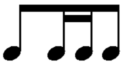	Presence in *lisimbu* repertoire is coded by l<br>Absence is coded by 0<br>Missing information is coded by ?
286	67	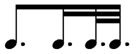	Presence in *mungala* repertoire is coded by 6<br>Absence is coded by 0<br>Missing information is coded by ?
287	68	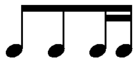	Presence in *lisimbu* repertoire is coded by l<br>Absence is coded by 0<br>Missing information is coded by ?
288	69	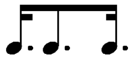	Presence in *ngodja* repertoire is coded by à<br>Absence is coded by 0<br>Missing information is coded by ?
289	4bi	Four pulsations cycle. Binary pulsation subdivision	Presence in *kenge* repertoire is coded by n<br>Presence in *makouma* repertoire is coded by s<br>Presence in *ngwata* repertoire is coded by 3<br>Presence in *mungala* repertoire is coded by 6<br>Absence is coded by 0<br>Missing information is coded by ?
290	4ter	Four pulsations cycle. Ternary pulsation subdivision	Presence in *berceuse* repertoire is coded by •<br>Presence in *mwiri* repertoire is coded by p<br>Absence is coded by 0<br>Missing information is coded by ?

291	6bi	Six pulsations cycle. Binary pulsation subdivision	Presence in *olamagha* repertoire is coded by j<br>Presence in *abire nkele* repertoire is coded by k<br>Presence in *ngoye* repertoire is coded by 5<br>Absence is coded by 0<br>Missing information is coded by ?
292	6ter	Six pulsations cycle. Ternary pulsation subdivision	Presence in *nzobi* repertoire is coded by 2<br>Presence in *mungala* repertoire is coded by 6<br>Absence is coded by 0<br>Missing information is coded by ?
293	8bi	Eight pulsations cycle. Binary pulsation subdivision	Presence in *nyembe* repertoire is coded by #<br>Presence in *berceuse* repertoire is coded by •<br>Presence in *ologo* repertoire is coded by a<br>Presence in *mbumba* repertoire is coded by b<br>Presence in *doka* repertoire is coded by d<br>Presence in *mbadi* repertoire is coded by e<br>Presence in *dijangu* repertoire is coded by r<br>Presence in *makouma* repertoire is coded by s<br>Presence in *mundukwe* repertoire is coded by t<br>Presence in *kunde* repertoire is coded by v<br>Presence in *laka* repertoire is coded by 4<br>Presence in *ngoye* repertoire is coded by 5<br>Presence in *mimbwiri* repertoire is coded by 9<br>Absence is coded by 0<br>Missing information is coded by ?
294	8ter	Eight pulsations cycle. Ternary pulsation subdivision	Presence in *berceuse* repertoire is coded by •<br>Presence in *ologo* repertoire is coded by a<br>Presence in *ngodja* repertoire is coded by à<br>Presence in *bubeyu* repertoire is coded by c<br>Presence in *langu* repertoire is coded by ç<br>Presence in *onkila* repertoire is coded by h<br>Presence in *ngungu tek* repertoire is coded by i<br>Presence in *abire nkele* repertoire is coded by k<br>Presence in *mavasa* repertoire is coded by l<br>Presence in *ibwema* repertoire is coded by o<br>Presence in *mujisi* repertoire is coded by q<br>Presence in *ngi* repertoire is coded by ù<br>Presence in *lisimbu* repertoire is coded by 1<br>Presence in *nzobi* repertoire is coded by 2<br>Presence in *ngoye* repertoire is coded by 5<br>Presence in *mungala* repertoire is coded by 6<br>Presence in *mimbwiri* repertoire is coded by 9<br>Absence is coded by 0<br>Missing information is coded by ?
295	12bi	Twelve pulsations cycle. Binary pulsation subdivision	Presence in *berceuse* repertoire is coded by •<br>Presence in *mbumba* repertoire is coded by b<br>Presence in *mbudi* repertoire is coded by f<br>Presence in *mundukwe* repertoire is coded by t<br>Presence in *mabandji* repertoire is coded by x<br>Presence in *minonze* repertoire is coded by y<br>Presence in *bwiti disumba* repertoire is coded by z<br>Presence in *lisimbu* repertoire is coded by 1<br>Presence in *laka* repertoire is coded by 4<br>Presence in *mungala* repertoire is coded by 6

			Absence is coded by 0<br>Missing information is coded by ?
296	12ter	Twelve pulsations cycle. Ternary pulsation subdivision	Presence in *boumba* repertoire is coded by $<br>Presence in *ngodja* repertoire is coded by à<br>Presence in *mavasa* repertoire is coded by l<br>Presence in *lisimbu* repertoire is coded by 1<br>Presence in *nzobi* repertoire is coded by 2<br>Presence in *mungala* repertoire is coded by 6<br>Presence in *mopfougou* repertoire is coded by 7<br>Presence in *wuya* repertoire is coded by 8<br>Absence is coded by 0<br>Missing information is coded by ?
297	16bi	Sixteen pulsations cycle. Binary pulsation subdivision	Presence in *bwiti misoko* repertoire is coded by *<br>Presence in *nyembe* repertoire is coded by #<br>Presence in *ologo* repertoire is coded by a<br>Presence in *mbadi* repertoire is coded by e<br>Presence in *mbudi* repertoire is coded by f<br>Presence in *issogha* repertoire is coded by g<br>Presence in *mujaja* repertoire is coded by m<br>Presence in *mujisi* repertoire is coded by q<br>Presence in *makouma* repertoire is coded by s<br>Presence in *kore* repertoire is coded by u<br>Presence in *kunde* repertoire is coded by v<br>Presence in *maboundi* repertoire is coded by w<br>Presence in *ngwata* repertoire is coded by 3<br>Presence in *laka* repertoire is coded by 4<br>Absence is coded by 0<br>Missing information is coded by ?
298	16ter	Sixteen pulsations cycle. Ternary pulsation subdivision	Presence in *berceuse* repertoire is coded by •<br>Presence in *boumba* repertoire is coded by $<br>Presence in *bubeyu* repertoire is coded by c<br>Presence in *deke* repertoire is coded by é<br>Presence in *bwiti koji* repertoire is coded by è<br>Presence in *mavasa* repertoire is coded by l<br>Presence in *ibwema* repertoire is coded by o<br>Presence in *ngi* repertoire is coded by ù<br>Presence in *lisimbu* repertoire is coded by 1<br>Presence in *nzobi* repertoire is coded by 2<br>Presence in *mungala* repertoire is coded by 6<br>Absence is coded by 0<br>Missing information is coded by ?
299	18ter	Eighteen pulsations cycle. Ternary pulsation subdivision	Presence in *ngodja* repertoire is coded by à<br>Absence is coded by 0<br>Missing information is coded by ?
300	20ter	Twenty pulsations cycle. Ternary pulsation subdivision	Presence in *nzokou* repertoire is coded by £<br>Absence is coded by 0<br>Missing information is coded by ?
301	24ter	Twenty four pulsations cycle. Ternary pulsation subdivision	Presence in *nzokou* repertoire is coded by £<br>Presence in *deke* repertoire is coded by é<br>Presence in *nzobi* repertoire is coded by 2<br>Absence is coded by 0<br>Missing information is coded by ?
302	irrégulier	Irregular pulsation subdivision	Presence in *bubeyu* repertoire is coded by c

			Presence in *doka* repertoire is coded by d<br>Presence in *mbadi* repertoire is coded by e<br>Presence in *ngoye* repertoire is coded by 5<br>Presence in *mungala* repertoire is coded by 6<br>Absence is coded by 0<br>Missing information is coded by ?
303	scale 1	Hexatonic with one semi-tone	Presence in *bwiti misoko* repertoire is coded by *<br>Presence in *nyembe* repertoire is coded by #<br>Presence in *berceuse* repertoire is coded by •<br>Presence in *nzokou* repertoire is coded by £<br>Presence in *ologo* repertoire is coded by a<br>Presence in *ngodja* repertoire is coded by à<br>Presence in *doka* repertoire is coded by d<br>Presence in *mbadi* repertoire is coded by e<br>Presence in *deke* repertoire is coded by é<br>Presence in *issogha* repertoire is coded by g<br>Presence in *abire nkele* repertoire is coded by k<br>Presence in *mavasa* repertoire is coded by l<br>Presence in *ibwema* repertoire is coded by o<br>Presence in *makouma* repertoire is coded by s<br>Presence in *kunde* repertoire is coded by v<br>Presence in *mabandji* repertoire is coded by x<br>Presence in *minonze* repertoire is coded by y<br>Presence in *bwiti disumba* repertoire is coded by z<br>Presence in *lisimbu* repertoire is coded by 1<br>Presence in *nzobi* repertoire is coded by 2<br>Presence in *laka* repertoire is coded by 4<br>Presence in *mungala* repertoire is coded by 6<br>Presence in *mimbwiri* repertoire is coded by 9<br>Absence is coded by 0<br>Missing information is coded by ?
304	scale 2	Hexatonic with two semi-tones	Presence in *ibwema* repertoire is coded by o<br>Presence in *makouma* repertoire is coded by s<br>Presence in *ngi* repertoire is coded by ù<br>Presence in *kunde* repertoire is coded by v<br>Presence in *lisimbu* repertoire is coded by 1<br>Presence in *nzobi* repertoire is coded by 2<br>Presence in *wuya* repertoire is coded by 8<br>Absence is coded by 0<br>Missing information is coded by ?
305	scale 3	Pentatonic anhemitonic	Presence in *berceuse* repertoire is coded by •<br>Presence in *ologo* repertoire is coded by a<br>Presence in *langu* repertoire is coded by ç<br>Presence in *onkila* repertoire is coded by h<br>Presence in *ngungu tek* repertoire is coded by i<br>Presence in *olamagha* repertoire is coded by j<br>Presence in *kenge* repertoire is coded by n<br>Presence in *nzobi* repertoire is coded by 2<br>Presence in *ngoye* repertoire is coded by 5<br>Presence in *mimbwiri* repertoire is coded by 9<br>Absence is coded by 0<br>Missing information is coded by ?

306	scale 4	Heptatonic with two semi-tones	Presence in *berceuse* repertoire is coded by •<br>Presence in *boumba* repertoire is coded by $<br>Presence in *ngodja* repertoire is coded by à<br>Presence in *mbumba* repertoire is coded by b<br>Presence in *bubeyu* repertoire is coded by c<br>Presence in *langu* repertoire is coded by ç<br>Presence in *doka* repertoire is coded by d<br>Presence in *mbadi* repertoire is coded by e<br>Presence in *deke* repertoire is coded by é<br>Presence in *bwiti koji* repertoire is coded by è<br>Presence in *mbudi* repertoire is coded by f<br>Presence in *mavasa* repertoire is coded by l<br>Presence in *mujaja* repertoire is coded by m<br>Presence in *ibwema* repertoire is coded by o<br>Presence in *mwiri* repertoire is coded by p<br>Presence in *mujisi* repertoire is coded by q<br>Presence in *makouma* repertoire is coded by s<br>Presence in *mundukwe* repertoire is coded by t<br>Presence in *kore* repertoire is coded by u<br>Presence in *ngi* repertoire is coded by ù<br>Presence in *minonze* repertoire is coded by y<br>Presence in *lisimbu* repertoire is coded by 1<br>Presence in *nzobi* repertoire is coded by 2<br>Presence in *ngwata* repertoire is coded by 3<br>Presence in *laka* repertoire is coded by 4<br>Presence in *ngoye* repertoire is coded by 5<br>Presence in *mungala* repertoire is coded by 6<br>Presence in *mimbwiri* repertoire is coded by 9<br>Absence is coded by 0<br>Missing information is coded by ?
307	scale 5	Pentatonic with one semi-tone	Presence in *ologo* repertoire is coded by a<br>Presence in *ngodja* repertoire is coded by à<br>Presence in *mbumba* repertoire is coded by b<br>Presence in *mavasa* repertoire is coded by l<br>Presence in *mwiri* repertoire is coded by p<br>Presence in *makouma* repertoire is coded by s<br>Presence in *kunde* repertoire is coded by v<br>Presence in *mabundi* repertoire is coded by w<br>Presence in *ngoye* repertoire is coded by 5<br>Presence in *mungala* repertoire is coded by 6<br>Presence in *wuya* repertoire is coded by 8<br>Presence in *mimbwiri* repertoire is coded by 9<br>Absence is coded by 0<br>Missing information is coded by ?
308	scale 6	Hexatonic with three semi-tones	Presence in *mundukwe* repertoire is coded by t<br>Presence in *lisimbu* repertoire is coded by 1<br>Absence is coded by 0<br>Missing information is coded by ?
309	scale 7	Tetratonic with one semi-tone	Presence in *berceuse* repertoire is coded by •<br>Presence in *mopfougou* repertoire is coded by 7<br>Absence is coded by 0<br>Missing information is coded by ?
310	scale 8	Tetratonic without semi-tone	Presence in *berceuse* repertoire is coded by •<br>Presence in *nyembe* repertoire is coded by #

			Presence in *ngwata* repertoire is coded by 3<br>Presence in *mimbwiri* repertoire is coded by 7<br>Absence is coded by 0<br>Missing information is coded by ?
311	scale 9	Nonatonic with six semi-tones	Presence in *dijangu* repertoire is coded by r<br>Absence is coded by 0<br>Missing information is coded by ?
312	scale 10	Pentatonic with two semi-tones	Absence is coded by 0<br>Missing information is coded by ?
313	scale 11	Octatonic	Presence in *nzokou* repertoire is coded by £<br>Presence in *mbumba* repertoire is coded by b<br>Presence in *ibwema* repertoire is coded by o<br>Presence in *mundukwe* repertoire is coded by t<br>Presence in *minonze* repertoire is coded by y<br>Presence in *ngwata* repertoire is coded by 3<br>Absence is coded by 0<br>Missing information is coded by ?
314	scale 12	Heptatonic with three semi-tones	Presence in *boumba* repertoire is coded by $<br>Presence in *mundukwe* repertoire is coded by t<br>Absence is coded by 0<br>Missing information is coded by ?
315	scale13	tritonic	Presence in *ngodja* repertoire is coded by à<br>Absence is coded by 0<br>Missing information is coded by ?
316	counterpoint	Plurilinear process	Absence is coded by 0<br>Presence is coded by 1<br>Missing information is coded by ?
317	partial counterpoint	Plurilinear process	Absence is coded by 0<br>Presence is coded by 1<br>Missing information is coded by ?
318	homorythmie	Plurilinear process	Absence is coded by 0<br>Presence is coded by 1<br>Missing information is coded by ?
319	responsorial	Song form	Absence is coded by 0<br>Presence is coded by 1<br>Missing information is coded by ?
320	antiphonal	Song form	Absence is coded by 0<br>Presence is coded by 1<br>Missing information is coded by ?
321	jodel	Vocal technique	Absence is coded by 0<br>Presence is coded by 1<br>Missing information is coded by ?
322	pseudo jodel	Vocal technique	Absence is coded by 0<br>Presence is coded by 1<br>Missing information is coded by ?
